# Natural Polymorphisms D60E and I62V Stabilize a Closed Conformation in HIV-1 Protease in the Absence of an Inhibitor or Substrate

**DOI:** 10.3390/v16020236

**Published:** 2024-02-02

**Authors:** Trang T. Tran, Gail E. Fanucci

**Affiliations:** Department of Chemistry, University of Florida, Gainesville, FL 32611, USA

**Keywords:** HIV-1 protease, conformational sampling, natural polymorphism, site-directed spin-labeling, DEER spectroscopy, EPR

## Abstract

HIV infection remains a global health issue plagued by drug resistance and virological failure. Natural polymorphisms (NPs) contained within several African and Brazilian protease (PR) variants have been shown to induce a conformational landscape of more closed conformations compared to the sequence of subtype B prevalent in North America and Western Europe. Here we demonstrate through experimental pulsed EPR distance measurements and molecular dynamic (MD) simulations that the two common NPs D60E and I62V found within subtypes F and H can induce a closed conformation when introduced into HIV-1PR subtype B. Specifically, D60E alters the conformation in subtype B through the formation of a salt bridge with residue K43 contained within the nexus between the flap and hinge region of the HIV-1 PR fold. On the other hand, I62V modulates the packing of the hydrophobic cluster of the cantilever and fulcrum, also resulting in a more closed conformation.

## 1. Introduction

HIV-1 infection occurs worldwide and still suffers from drug resistance and virological failure [1]. Treatment strategies for HIV-1 infection include a mixture of classes of drugs that target essential components of the HIV-1 lifecycle and are referred to as highly active antiretroviral therapy (HAART) [2]. Although this approach is seeing great success and many people today live extended lifetimes, drug resistances emerge to many of the classes of drugs in HAART [3,4,5,6,7,8].

**Figure 1:**
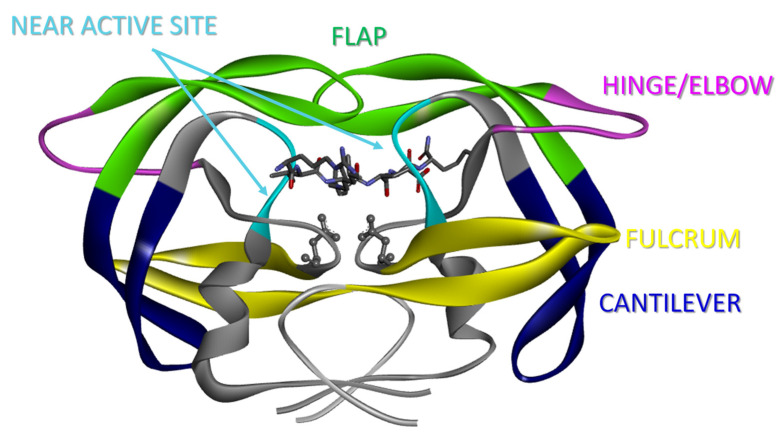
Ribbon diagram of a highly drug-resistant HIV-1 PR, PRS17, in complex with substrate analog CA-P2 [PDB ID 6O5X], showing the flaps closed around the substrate analog/inhibitor in the active site, with active site aspartic acid residues D25 and D25′ rendered in ball and stick format. Several structural regions discussed within are rendered in varying colors, such as the “flap region” (residues 43–59) in green, the “hinge/elbow region” (residues 35–42) in bright pink, the “fulcrum region” (residues 9–24) in yellow, the “cantilever region” (residues 60–75), and the “near-active site region” (residues 80–84) in cyan.

HIV-1 protease is a relatively small homodimeric (99 amino acids in each monomer) aspartic acid protease [9,10] essential to the infectious lifecycle of the HIV-1 virus, the causative agent of Acquired Immunodeficiency Syndrome (AIDS) [11,12]. HIV-1 PR cleaves the polypeptides Gag and Gag-Pol, allowing for the assembly of mature infectious virions [13]. Current FDA-approved protease inhibitors (PIs) bind to the active site of HIV-1 PR [3,4,5,14] and “lock” the flaps in a closed conformation with the inhibitor bound in the active site pocket (Figure 1). Access to the floor of the active site is gained upon opening of two *β*-hairpins, termed “flaps” in HIV-1PR [15,16,17,18]. The motion of the flaps plays a fundamental role in the activity and function of this protein [15,16,18,19,20,21,22,23,24,25,26,27,28,29,30]. Numerous structural and computational studies of HIV-1 PR in an unbound state show the flaps in a conformation that allows access to the active site [16,17], and kinetic studies indicate a conformational change of flap opening before substrate can be processed [29]. These open states have been described as semi-open [10,16,17,31,32], wide-open [16,33,34,35], and tucked/curled-open [36,37]. Wide-open and curled-open conformations are being increasingly observed for HIV-1 PR that has accumulated drug-pressure-selected mutations [8,30,36,38,39]. Given that the current FDA-approved inhibitors for HIV-1 PR have been designed based upon the substrate-induced closed transition state of the sequence of the dimeric protein found predominantly within North America and Europe (also known as subtype B), the wide-open and curled-open conformations may be considered as conformational targets for rational drug design [8,34].

Patterns of drug-pressure-selected mutations HIV-1 PR with respect to specific PI regimens (Stanford HIV Database) has shown amino acid changes at approximately half of the amino acid site positions [5,40,41], with 5 to 15 mutations in the PR gene being typical for resistant patients, with higher numbers of mutations (10–20) necessitated for resistance to Tipranavir (TPV) [7,42,43,44] and Darunavir (DRV) [45,46]. Additionally, there is great genetic diversity in HIV-1 viruses spread throughout the world [47], where random mutations generate natural polymorphisms (NPs). Many of these NPs correspond to accessory drug-pressure-selected mutations in HIV-1 PR [48]. Although HIV-1 PR NPs only moderately impact enzyme activity and viral fitness, their presence has been shown to give rise to enhanced drug resistance [49,50,51,52]. Thus, by obtaining a better understanding of these alternate conformations as well as “hot spots” that can help induce a closed-like state, this work adds to a growing field of novel ways to target HIV-1 PR and drug resistance [38,48,53,54,55,56,57,58,59,60,61,62,63,64,65]. These concepts are also being explored in drug development against other proteases in emerging diseases [66,67,68].

Pulsed EPR investigations can be used to interrogate the conformational sampling of proteins [69,70,71,72,73,74,75]. Our lab has utilized this approach to study the conformational landscape of HIV-1 PR, showing that inhibitor and substrate binding to HIV-1 PR induce a conformational shift to the closed state [18,26,27,76,77,78,79,80,81,82]. These investigations have also shown that drug-pressure-selected mutations and natural polymorphisms alter the fractional occupancy of four ascribed conformational states of HIV-1 PR, namely closed, semi-open, wide-open, and curled/tucked [27,81,82]. Insights regarding how drug-pressure-accumulated mutations lead to multidrug resistance have been gleaned from detailed analysis of pulsed EPR results that characterize changes in the conformational sampling of HIV-1 PR [25,77,79].

**Figure 2:**
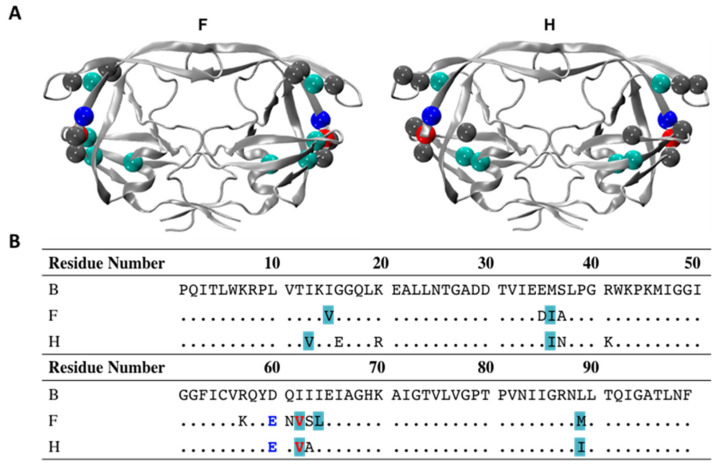
Comparison of HIV-1 PR sequences of subtypes B, F, and H. (**A**) Ribbon diagram of the closed conformation (PDB ID 2PBX) showing the locations of natural polymorphisms (NPs) and (**B**) sequence alignment comparisons. All NPs are represented as spheres, with D60E in royal blue, I62V in red, hydrophobic core residues in teal, and other NPs in gray. Hydrophobic core residues are underlined in the PI-naive subtype B sequence.

Of particular interest to us was the observation that the closed-conformation of HIV-1 PR, which is typically induced by substate/inhibitor binding, became dominant over the semi-open conformation for subtypes F and H in the absence of inhibitors [80]. We also observed the dominance of the closed conformation with single amino acid substitutions A71V [25] and L63P [83] contained within subtype B. Here we report insights, from both pulsed EPR and computational studies, into the effects that natural polymorphisms D60E and I62V have on the PI-naive conformational landscape of subtype B HIV-1 PR. These residues were chosen based upon examination of the amino acid sequences of subtypes F and H, revealing their common NPs: D60E and I62V [80]. Figure 2 shows the sequence of the F and H variants and highlights the location of these substitutions in the cantilever of HIV-1 PR.

## 2. Materials and Methods

### 2.1. Cloning and Site-Directed Mutagenesis

Site-directed mutagenesis reactions using the Quick-change kit were performed on a pET-23a plasmid encoding the LAI subtype B sequence shown in Figure 2 to generate plasmids encoding the D60E, I62V, and D60E/I62V variants. The subtype B sequence used was described previously and was codon-optimized and cloned between the *Nde*I and *Bam*HI restriction sites [18,76,84]. All constructs for experimental studies contained the following substitutions: D25N to inactive HIV-1PR to aid in protein stability by limiting autoproteolysis [14,78,81], Q7K, L33I, and L63I as stabilizing mutations that are commonly used within the subtype B sequence of an active enzyme to aid in preventing autolysis of HIV-1PR [84,85], C67A and C95A for the removal of native cysteine residues [18,86], and K55C for site-specific spin labeling for DEER experiments, which has been shown not to alter enzyme activity [18,87]. The fidelity of each HIV-1 PR gene was confirmed by Sanger DNA sequencing (ICBR Genomics Facility, University of Florida).

### 2.2. Expression, Protein Purification, and Spin Labeling

Protein expression, purification, and spin labeling were carried out using protocols previously described [39,78,79,80,83,85], with the pH of the inclusion body resuspension buffer adjusted to one unit above the isoelectric point (pI) of each construct, which for all three variants studied here is 9.39. The spin label (1-oxyl-2,2,5,5-tetramethyl-d3-pyrrolidine-3-methyl)methanethiosulfonate (MTSL) (Santa Cruz Biotechnology, Santa Cruz, CA, USA) was freshly dissolved into ethanol (~1 mg into 100 µL) and an appropriate volume added to achieve a 4:1 SL:P molar excess during the labeling reaction, which was carried out at 4 °C (to prevent protease precipitation) in the dark overnight. The protein precipitate was removed by centrifugation (17,210× *g*, 25 °C). The excess free spin label was removed, and protein was buffered to 2 mM NaOAc, pH 5.0, using a HiPrep 26/10 desalting column (GE Healthcare, Chicago, IL, USA).

### 2.3. DEER Experiments and Data Analysis

HIV-1 PR samples were concentrated to 140 μM and buffer exchanged to 20 mM d_3_-NaOAc/D_2_O, pH 5.0, using centrifugal membrane concentrators (Millipore, Billerica, MA, USA). The protein sample was then transferred to a 4 mm quartz EPR tube (Norell, Marion, NC, USA) and flash-frozen in liquid nitrogen. The sample was then immediately inserted into the ER 4118X-MD-5 dielectric ring resonator. DEER experiments were carried out at 65 K using a four-pulse DEER sequence [69,72]. The DEER modulation curves were background-corrected and converted to distance distribution profiles via Tikhonov regularization using DeerAnalysis [88]. To obtain the fractional occupancy of conformational states of HIV-1PR, we deconstruct the DEER distance profiles into a linear superposition of Gaussian-shaped populations using DEERconstruct (a program developed in our laboratory) [89,90] to represent the conformational states of curled/tucked, closed, semi-open, and wide-open states.

### 2.4. Molecular Dynamics Simulations

MD simulations were performed using the HiPerGator2.0 supercomputer at the University of Florida. The starting coordinates of HIV-1PR LAI were taken from PDBID: 1HHP and converted to a dimer [17]. The amino acid substitutions described above and outlined in Figure 1 that are not present in the 1HHP sequence that are included in simulations are the following: Q7K, D25N, L33I, C67A, and C95A for subtype B. The amino acid substitutions were incorporated using the *tleap* module of the AMBER suite of programs. Starting structures for HIV-1PR D60E, I62V, and D60E/I62V were generated from the above subtype B sequence. The starting structure for HIV-1PR bound to Darunavir for simulations of a closed conformation was taken from PDBID: 3BVB [14]. Simulations were set up using the procedure previously published [91,92,93,94,95]. All molecular dynamics (MD) simulations employed the *ff99SB* forcefield and the AMBER suite of programs [96,97]. Prior to starting simulations, each protein system was run through the H++ protonation state server [98] to model the charged amino acids based on their protonation states [99]. Each system was then solvated in a rectilinear periodic box of explicit SPC/E water molecules set at pH 6.8 [100], with the box extending 8 Å from the solute. Counterions were added to net-neutralize the systems. For all simulations, the proteins went through a five-stage minimization followed by a two-stage equilibration prior to running production. In the first four stages of minimization, the following were allowed to be energy minimized in the following order: all water molecules and counterions, all hydrogen atoms, side chain groups, and backbone amide groups. In the last stage of minimization, the entire system, including proteins, water molecules, and counterions, was allowed to be energy minimized through a progressive release of constraints. The systems were slowly heated to 300 K over 100 ps for the canonical ensemble. In the last stage of equilibration, 1 ns of MD was performed at 300 K for an isothermal and isobaric (NPT) ensemble. The simulations were then run at 300 K in the NPT ensemble. A Langevin thermostat with a 2 ps^−1^ collision frequency was employed to maintain the temperature of the system [101,102]. The SHAKE algorithm [103] was used to restrain hydrogen bond lengths, and the particle mesh Ewald method was used to calculate long-range electrostatic interactions. A time step of 2 fs was utilized. A total of 1.6 μs of MD data were collected for each system. MD analyses were performed using the *ptraj* tool of AMBERTools [96].

## 3. Results and Discussion

### 3.1. D60E and I62V Shift the HIV-1 PR Conformational Sampling to Closed-like States

**Figure 3:**
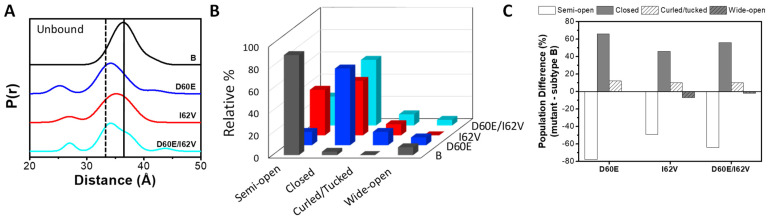
Conformational sampling of HIV-1 PR in the unbound state. (**A**) Stack plot of DEER distribution probability profiles. Data are vertically offset for clarity and probability normalized to 100%. The dash line and solid line are placed at 33 Å (closed conformation) and 36 Å (semi-open conformation), respectively. (**B**) Relative populations for each HIV-1PR construct via deconstruction analysis of the DEER distance profiles, where the flaps are assigned four conformations with the following distances between spin-pairs: curled/tucked (24–30 Å), closed (~33 Å), semi-open (~36 Å), and wide-open (40–45 Å). (**C**) Difference in population analysis relative to subtype B. Data for B is the same as reported within ref. [76]. Full data analysis is provided in supporting information (Figures S1–S7).

To characterize the impacts of these NPs on the conformational landscape of the unbound enzyme, DEER analysis was performed for WT, the two single and the double substituted HIV-1 PR constructs, to obtain the K55C-MTSL interspin distance distribution probability profiles given in Figure 3. Because HIV-1PR is a homodimer, the incorporation of a single spin-label site generates a spin-pair for distance measurements that can be used to describe the conformational landscape of HIV-1PR [18,27,39,89]. Overall, D60E is found to alter the conformational sampling more dramatically than the I62V substitution. The most probable K55C interspin distance in subtype B is 36.2 ± 0.2 Å, which corresponds to a predominant semi-open conformation (Figure 2A) [76]. D60E shifts the most probable interspin distance to 34.0 ± 0.2 Å, like the effects we observed previously for other single amino acid substitutions, L63P [95] and A71V [25] (Table 1). In contrast, a less dramatic shift in the most probable distance is observed for I62V, with a most probable interspin distance of 35.2 ± 0.2 Å, which occurs between the semi-open and closed distances. When both substitutions are included, the conformational landscape contains a marked bimodal shape with two major peaks at 34.2 ± 0.2 Å and 36.5 ± 0.2 Å.

The DEER conformational landscape represents the summation of the four previously mentioned HIV-1 PR conformations, specifically the inhibitor-bound closed state, the semi-open state, the wide-open state, and the curled open/tucked flap states [18,27,39,89]. Through several investigations, we identified the distances between the K55 spin pairs of each of the four conformational states as 25–30 Å for a flap-curled open/tucked conformation, 33 Å for the closed-conformation, 36 Å for the semi-open conformation, and >40 Å for the wide-open conformations [25,27,79,80,95]. The fractional occupancy of a given conformation is obtained by fitting the DEER distance profile to a linear combination of Gaussian-shaped populations that corresponds to the four above-described conformations of HIV-1 PR [89,90]. The Gaussian distribution diagrams for the DEER distance profiles in Figure 3A are given in Figures S1–S7. The relative percentages of each Gaussian population, a.k.a. population analysis, are shown in Figure 3B and Table 1, with the relative differences in population to subtype B shown in Figure 3C. Analysis reveals that all constructs have greater occupancy of the closed conformation (65%, 49%, and 59% for D60E, I62V, and D60E/I62V, respectively) compared to the subtype B conformational landscape and demonstrate that the closed conformation is the dominant conformation for each construct.

We previously studied the impact of other cantilever mutations. The mutation L/I63P substitution also shifts the conformation to the closed state (fractional occupancy 57%), increases backbone rigidity, and induces a closed structure. The flaps demonstrated the handedness of an inhibitor-induced closed structure [PDB ID: 5T84] when crystallized in the absence of any substrate analog or inhibitor [95]. Additionally, the single substitution A71V also dramatically shifts the conformational sampling to a predominantly closed state (67% fractional occupancy) [25]. The fractional occupancy of the wide-open state did not change for D60E and D60E/I62V relative to B, whereas no population was observed for wide-open conformation in I62V. Furthermore, DEER distance profiles for constructs had a statistically relevant population (within the SNR of the data, Supporting Information) below 30 Å (10–12%) that we assigned to a curled/tucked conformation [81,82,95], displaying a higher population than observed in subtype B (4 ± 4%). Although the increase in the fractional occupancy of the curled/tucked conformation was less than that seen for the closed conformation, results reveal that D60E and I62V substitutions lead to an increase in flap curling. Additionally, upon complexation with DRV, these populations are retained within the “bound” distance profiles (Figure S1–S7).

### 3.2. I62V Participates in the Hydrophobic Siding Mechanism

As secondary mutations arise in response to binding with PIs, they are generally located in the core of HIV-1 PR away from the catalytic pocket, yet they may still indirectly facilitate or contribute to conformational changes [30,80,82,104,105]. The core of HIV-1 PR, i.e., the fulcrum and cantilever, contains 19 hydrophobic residues outside of the catalytic pocket [105,106,107,108]. Of these 19 hydrophobic residues, nine are isoleucine, which has more possible conformations than other hydrophobic amino acids. When a mutation in the core arises, it may disrupt the hydrogen-bonding network or the van der Waal interaction network within the core. With many isoleucine residues in the core, the side chain of a residue can change its conformation, causing the hydrophobic surface to slide from one hydrophobic residue to another by exchanging van der Waal contact, which in turn facilitates conformational change at minimal energetic expense [106].

**Figure 4:**
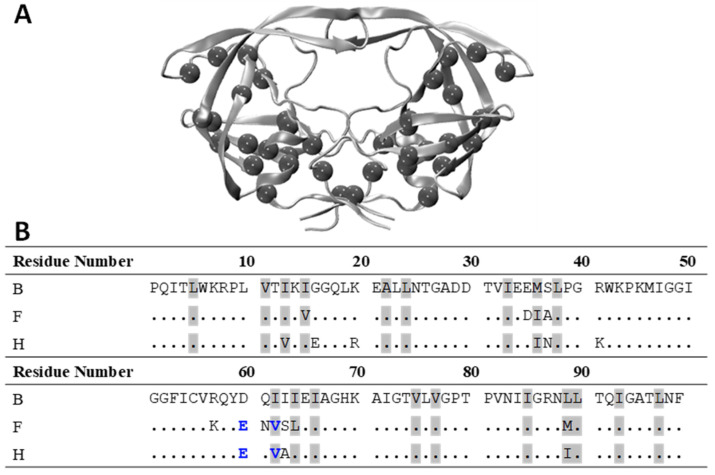
Hydrophobic core residues. (**A**) Ribbon diagram of subtype B HIV-1 PR with hydrophobic core residues shown in spheres. (**B**) Sequence of subtypes F and H compared to subtype B with hydrophobic core residues highlighted in gray as well as D60E and I62V mutations shown in blue.

The residues participating in the hydrophobic sliding mechanism are shown in Figure 4. Structurally, sites 63, 71, 62, 64, 66, 89, and 93 interact with one another within the hydrophobic core cluster [106]. Subtypes F and H variants both have four NPs in the hydrophobic core, one of which is I62V. Mutation of the hydrophobic core residues is more likely to affect the dynamic properties of HIV-1 PR and inhibitor binding but not substrate binding [106,108]. Thus, it is reasonable that I63V substitutions can modulate the hydrophobic packing/sliding in this cluster, resulting in an induced shift in the stability of the conformational landscape. The combination of D60E and I62V generates an averaged impact on the shift to the closed conformation. The offsetting effects of the double substitution are discussed further below.

### 3.3. Root-Mean-Square Deviation during Simulations

**Figure 5:**
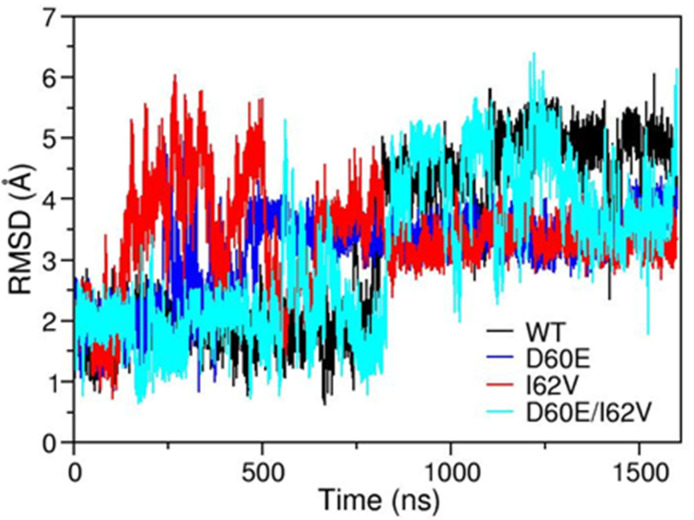
Root mean square deviation of the flap region residues 43–59 of WT, D60E, I62V, and D60E/I62V relative to the equilibrated 1HHP structure.

MD simulations from HIV-1PR subtype B (labeled as WT), D60E, I62V, and D60E/I62V were performed to further study the effects these amino acid substitutions have on the dynamics and conformational flexibility of HIV-1 PR. Figure 5 shows the calculated root-mean-square deviation (RMSD) of the backbone in the flap regions (residues 43–59) of the four constructs over the course of the simulation. The RMSD pattern for WT and D60E/I62V is similar, with values near 2 Å for the first 800 ns and a jump to 4.5–5 afterwards. Although similar, the D60E/I62V has RMSD values that fluctuate more frequently than do those for WT. Specifically, there is a jump in RMSD from 2 Å to 5.5 Å around 600–700 ns, followed by a drop in RMSD of 1.5 Å around 700–800 ns. RMSD of the D60E/I62V then increases to ~5 Å, like the behavior observed in WT, but then has additional fluctuations between 2–5 Å over the 800–1300 ns range. Between 1300 and 1550 ns, the RMSD for D60E/I62V decreased from 5 Å to 3.5 Å, like the behavior seen for D60E/I62V in this period. Finally, the RMSD value increased to 5 Å in the last 50 ns. The RMSD of both single mutants, D60E and I62V, fluctuates largely from 1–6 Å in the first 500–600 ns and stabilizes to a value of ~3.5 Å during the last 900 ns, which is different from WT.

### 3.4. D60E Mutation Facilitates a Salt Bridge Interaction

**Figure 6:**
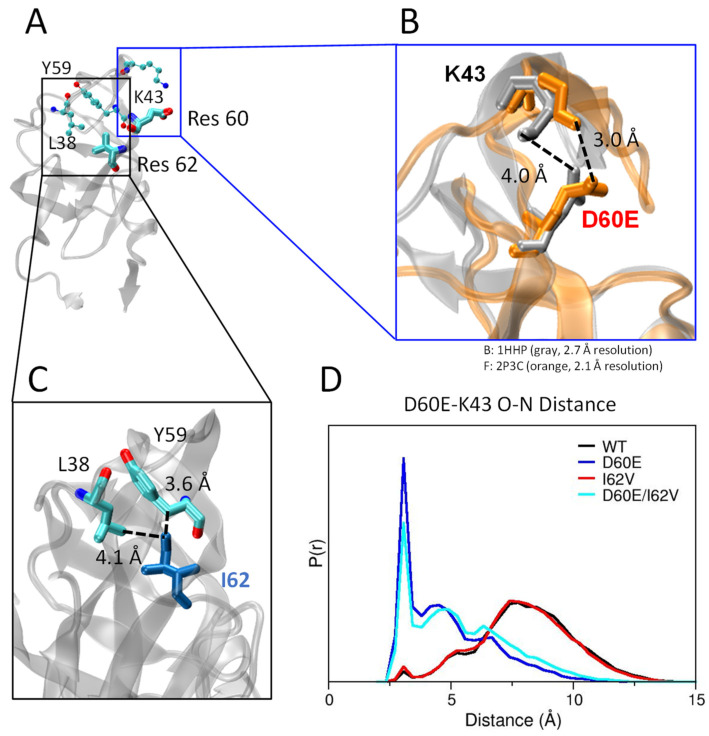
(**A**) Ribbon diagram (PDBID 2P3C) in gray showing a side view of HIV-1 PR, highlighting interactions of residues 60 and 62 with the local environment. D60E and I62V are rendered in stick format, whereas other residues are shown as sticks and balls. (**B**) Overlay of crystal structures of subtype B (1HHP, silver) and subtype F (2P3C, orange) showing D60E to K43 distance. Residues D60 and K43 in subtype B are shown as silver sticks and have an N-O distance of 4.0 Å. Residues E60 and K43 in subtype F are shown as orange sticks and have an N-O distance of 3.0 Å. (**C**) Interaction of I62 with L38 and Y59 in 1HHP. The residues are shown as sticks, with I62 shown in blue. (**D**) Probability distribution profile for N-O side chain distances of D/E60 and K43 indicative of salt bridge interactions determined from MD trajectories.

Site D60 within HIV-1PR sits in the cantilever region (residues 60–75), as shown in Figure 1 and Figure 6A. When comparing the crystal structures of PI-naïve subtype B (i.e., *WT*) and subtype F (which contains the D60E substitution, Figure 6B), one can readily see a salt bridge between D60E-K43 in the subtype F structure (3.0 Å) that is altered in WT (4.0 Å) due to the shorter length of Asp (D) compared to GLU (E). The D60E-K43 bridge connects the cantilever (D60E) to the flap/hinge nexus (site K43) in HIV-1PR. It has been shown in the literature that salt bridge interactions in the hinge region are important and can affect mobility as well as the conformational landscape of HIV-1 PR. One such salt bridge interaction that was previously studied in our laboratories is E35D-R57K, which connects the flap region (R57K) to the elbow/hinge region (E35D), which we showed can modulate the relative population of the curled/tucked flap conformation [81,82]. Additionally, Schiffer and colleagues demonstrated the impact of reversible disulfide linkages between the elbow/hinge and flap (L38C/G16C) and the cantilever and flap (E65C/G16C) [108]. These crosslinking studies showed that restriction of motion by crosslinking the flap to the elbow/hinge diminished catalytic activity (~150 fold lower than WT), whereas the linkage between the cantilever and flap did not impact catalytic activity (~1× WT) [108].

Analysis of the MD simulation runs shows that the relative population of this salt bridge is significantly increased in WT when D60E is included. Analyses of MD trajectories for WT and I62V reveal a broad D60-K43 N-O distance, spanning from 3–10 Å with a major peak at 7.5–8.5 Å and a smaller shoulder at 5.2 Å (Figure 6D). The presence of a very small peak at 3.0 Å indicates only a slight tendency for a salt bridge interaction in these two constructs. This interaction is observed within the WT crystal structure (PDB ID 1HHP), but only moderately populated when molecular dynamics are included, which highlights the potential drawbacks of looking exclusively at static crystal structures that may stabilize conformations due to crystal packing. In contrast, simulation runs for constructs that contain the D60E substitution (D60E and D60E/I62V) show a dramatic increase in the probability density at 3.0 Å, indicating the D60E-K43 salt bridge is much more favorable when residue 60 is altered to Glu. Additionally, the 7.5–8.5 Å peak seen in WT and I62V has significantly reduced, and the shoulder at 5.2 Å shifts to shorter values of 4.5 Å in the presence of D60E.

The intensity of the 3.0 Å peak in the D60E/I62V simulation is lower than that in WT. D60E/I62V also shows a slight shift of the 4.5 Å peak to higher distances and a higher intensity for distances greater than 5.0 Å. In WT, I62 Cα has van der Waals contacts with Y59 Cα and L38 Cα1 at 3.6 Å and 4.1 Å, respectively (Figure 6C). When mutated to Val, the side chain of residue 62 is shortened by one carbon bond, and interactions with L38 and Y59 may be lost, possibly resulting in more flexibility in the hinge region, which may explain the lowered intensity observed for the 3.0 Å peak in I62V simulations. Perhaps the addition of the I62V mutation to the D60E mutation slightly destabilizes the D60E-K43 salt bridge to retain flap flexibility and help restore the semi-open population, as seen in DEER results (Table 1).

### 3.5. Distance Measurements for Conformational Assignment in Simulations

**Figure 7:**
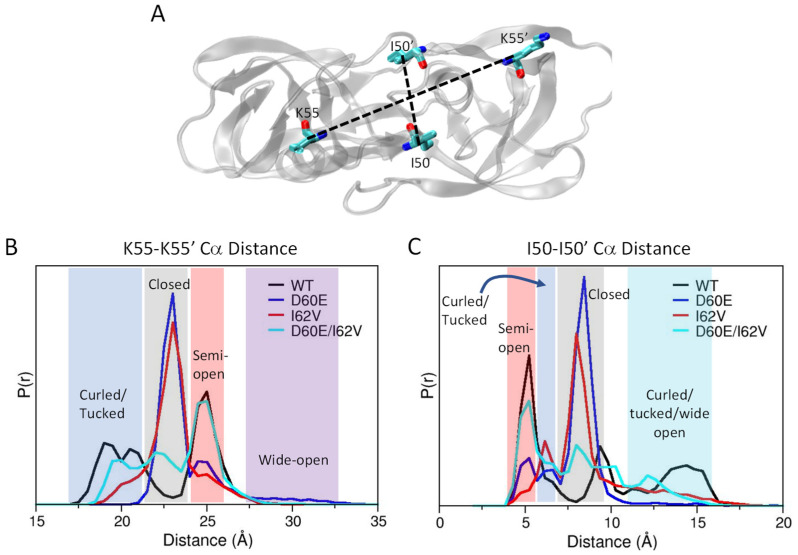
(**A**) Top view of HIV-1 PR (PDB ID 1HHP) with residues I50, I50′, K55, and K55′ shown as sticks and dash lines showing the distances measured. (**B**) Flap distance (K55-K55′) and (**C**) flap tip (I50-I50′) distances as probes for conformation in simulations. Grey coloring indicates distances corresponding to a closed flap conformation, red coloring indicates distances corresponding to the semi-open conformation, blue coloring indicates distances corresponding to a curled/tucked open flap conformation, purple coloring indicates a wide-open flap conformation, and the teal coloring indicates distances consistent with both the wide-open and curled/tucked open flap conformations.

To better define conformations probed via the flap K55R1-K55R1′ distance distribution profiles seen with DEER spectroscopy, we evaluated the K55C-K55C′ Cα distance together with the I50-I50′ Cα distance of the flap tips to generate distribution profiles to assess and describe conformations sampled during the MD trajectories. The locations of these residues in the flaps of HIV-1PR are shown in Figure 7A. With regards to the K55-K55′ Cα (Figure 7B) distance probability profile, WT protease displays three distances, with a major peak at 25 Å (assigned as semi-open) and two minor peaks at 19 Å and 21 Å, which we label a curling or tucking of flaps that represent an open-like state [25,79]. When each single amino acid substitution is incorporated, there is a dramatic change observed in the distance distribution profile compared to WT. Specifically, a new major peak appears at 23 Å (assigned as closed) that was not seen in WT, with diminished or no intensity for peaks at 25 Å, 21 Å, and 19 Å. When D60E and I62V are combined, the 25 Å peak regains similar intensity as observed in WT but also retains some probability density at 23 Å and 19–20 Å. We assign these K55-K55′ Cα peaks to the four conformations of HIV-1 PR seen in DEER data analyses and X-ray structures as: curled/tucked open (17–21 Å), closed (23 Å), semi-open (25 Å), and wide-open (27.5–33 Å).

Furthermore, I50-I50′ Cα distance distribution profiles (Figure 7C) show a similar effect. Specifically, the single amino acid substitutions markedly alter the distribution profile of WT, with diminished probability at 5 Å (the major peak observed for WT) and the growth of new probability peaks at 9.4 Å and 8.5 Å for D60E and I62V, respectively, that are absent in WT data. The combination of the two substitutions again results in an averaged distance distribution profile where the probability at 5 Å grows towards WT values but where D60E/I62V retains the new distance of ~9 Å.

**Figure 8:**
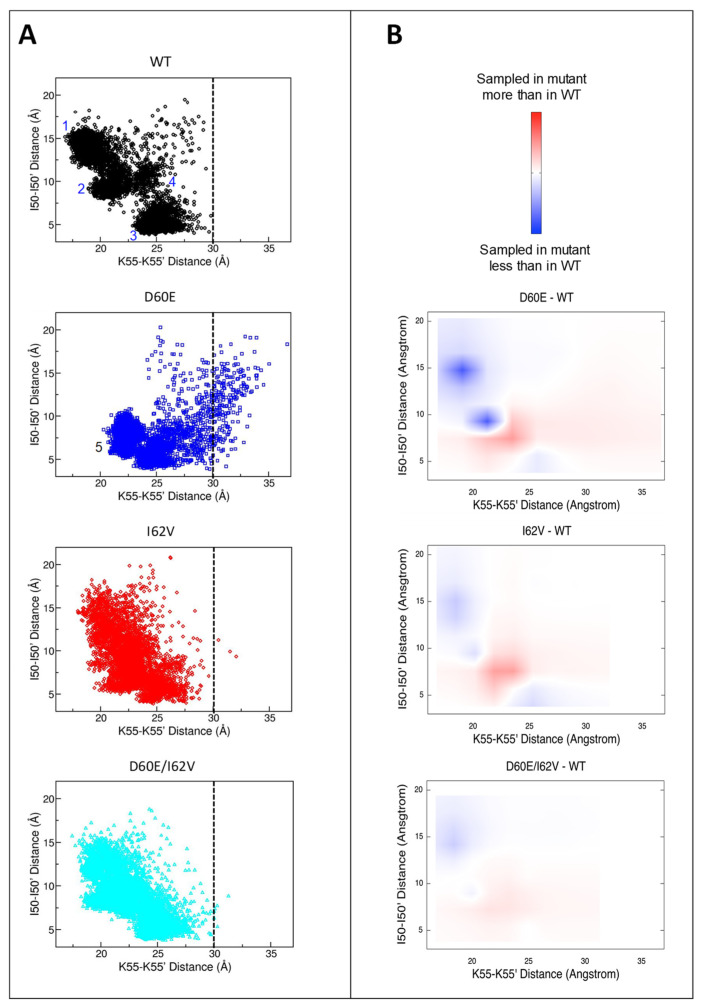
Conformational landscape of HIV-1 PR during simulations. (**A**) Double distance plots representing the conformational landscape of WT, D60E, I62V, and D60E/I62V in MD simulations determined from the distances of each conformer within the simulation. Labels 1 and 2 are assigned to a flap curling/tucking open-like state, with label 3 representing the semi-open conformation, label 4 representing an unknown conformational population and label 5 representing a flap closing—as would be induced by inhibitor or substrate binding. (**B**) Difference plots between the double distance plots of mutants and WT.

The conformations related to I50-I50′ Cα distances are assigned based on a survey of the I50-I50′ Cα distance from various crystal structures of HIV-1 PR [16,34,35,49,109,110] (Table S1), and our MD trajectories are discussed more below, including that of WT protease bound to Darunavir (Figure S8), which is known to stabilize a closed structure [14]. When protease is in an inhibitor-bound closed conformation, the flap tips cross over one another. As the enzyme opens to a semi-open state, the distance between sites I50 and I50′ in the tips is closer to one another than when the inhibitor is bound in the closed state, resulting in a shortened distance between the I50-I50′ Cα atoms. The flap and flap tips move furthest from each other in the wide-open conformation. Therefore, we assign these I50-I50′ Cα peaks to the HIV-1 PR conformations: closed (8.5 Å), semi-open (5 Å), and wide-open (13–16 Å). When the flaps curl or tuck open, the I50-I50′ Cα distance increases; therefore, the population observed for >11 Å is assigned as a combination of the open-like states. We also propose (see double distance plots in Figure 8) that the flap tip distance of 6 Å for I50-I50′ Cα also corresponds to curled/tucked conformation. We computationally determined the closed state MD K55-K55′ Cα and I50-I50′ Cα distance distribution profiles from analysis of the MD trajectories of WT-bound to DRV, in agreement with ~8 Å and ~6 Å for the I50-I50′ Cα distance distribution of the closed and curled/tucked conformations, respectively. For the DRV-bound HIV-1PR simulations, the K55-K55′ Cα is centered at 22.8 Å (Figure S8). This agrees with the K55-K55′ Cα data: WT assembles different conformations. When the D60E mutation is introduced, the conformation of the PR is shifted to closed. The I62V mutation also shifted conformation to closed, but to a lesser extent. When both mutations are introduced, the conformations seen in WT are regained while also retaining a portion of the closed conformation.

### 3.6. Alterations in the Conformational Landscape by Double Distance Measurement

Even though Figure 7 shows probability distance profiles of flaps and flap tips for conformers sampled throughout the simulation, time information is not captured within Figure 7, which makes it difficult to correlate these distances and assign conformations. Double-distance plots showing both distances, flaps (K55-K55′), and flap tips (I50-I50′), in the same snapshot, can better reveal the conformational landscape of HIV-1 PR throughout the simulation (Figure 8). Figure 8A shows WT assembled predominantly from three different conformational populations represented by three different areas labeled 1–3, with area 3 representing the semi-open conformation. There is also a smaller area labeled 4, representing a fourth conformational population with a much lower probability compared to the other three conformations. During the MD runs for WT, the wide-open conformation was not sampled. Instead, access to the active site appeared due to the flap curling/tucking as evidenced by populations 1 and 2 with short K55-K55′ flap distances and I50-I50′ flap tip distances spanning 6 to 18 Å.

The conformational landscape of the D60E single mutant differs dramatically from WT. D60E sampled larger flap distances (K55-K55′) above 30 Å, which was not the case for WT. In D60E, conformations 1 and 2 are absent, with the appearance of a new conformational population represented by area 5. This area 5 had a flap distance of 21–25 Å and a flap tip distance of 6–9 Å, which we assigned to the closed conformation as these distances were observed in the MD trajectories of the DRV-bound subtype B (Figure S8). This result is consistent with the experimental DEER data, where the D60E substitution increases the closed conformation percentage in HIV-1 PR.

The double distance plot of I62V shows a similar ensemble pattern compared to WT. The distance pairs, however, spread a wide range of distance combinations within the conformational landscape instead of assembling into more defined areas, as seen in the WT and D60E single mutants. Nevertheless, closed conformation represented by area 5 was also present, as evidenced by data points at 21–24 Å for flap distance and 6–9 Å for flap tip distance (Figure 8A). The blue density in Figure 8B at the position of area 1 showed that the conformation represented by area 1 was decreased to a lesser degree compared to the difference plot of D60E-WT (Figure 8B). In other words, the conformation for area 1 in I62V was decreased to give rise to the closed conformation represented by area 5.

A similar conformational ensemble to the I62V single mutant was observed for the D60E/I62V double mutant. The conformational ensemble of D60E/I62V was spread over a wide range of distance combinations, even more like WT than I62V. In contrast to the double distance plot of both single mutants, area 5 was not clearly observed in the double distance plot of D60E/I62V. The data points for areas 5 (increase in closed conformation) and 1 (decrease in flap curling/tucking) in Figure 8B were even less intense compared to the I62V single mutant. These changes in the conformational landscape of the double mutant seemed to be minimal, and the overall conformational landscape is shown to be very similar to that of WT.

### 3.7. Average Structure during Simulations

**Figure 9:**
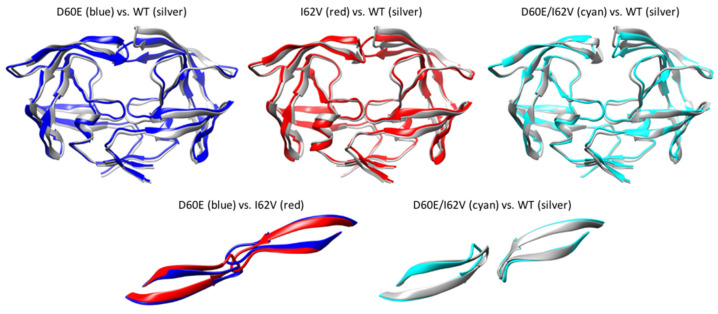
(**Top row**) Front view of the average structure of mutants compared to WT during MD simulations. (**Bottom row**) Top view of the average structure showing the handedness of WT and mutants.

The average coordinates of each construct over the course of 1.6 μs simulations were also calculated, and the average structures are shown in Figure 9. In general, the average structures did not have large variation in other regions of the PR except for the flap region, especially flap tips. The average structure of WT is in semi-open conformation, with one flap raised higher than the other flap and slightly curled flap tips. On the other hand, both single mutants adopted the closed conformation on average, with their flap tips rather straight (Figure S9). The average structure of D60E/I62V has more in common with the average structure of WT. On average, the double mutant adopted more of a semi-open conformation as well as curled flap tips, like the average structure of WT. The double mutant had one flap slightly raised higher than that of either single mutant but not as high as the WT. The combination of both mutations resulted in an average structure halfway between WT and single mutants. The flap tips of double mutants were also more curled compared to the flap tips of WT.

The flap-handedness of WT is the same as in the starting structure and crystal structure of B (PDB ID: 1HHP). An inversion in flap-handedness was observed for both single mutants. Not only did the double mutant adopt similar flap conformation as WT on average, but it also had the same flap-handedness as WT. This agrees with our hypothesis that the I62V mutation serves as a compensatory mutation in the presence of the D60E mutation to regain conformation of WT. Figure 9 also shows that the flap conformation as well as the flap-handedness of WT and the double mutant are similar in their starting structure. On the other hand, the flap conformation and flap-handedness of the single mutants are similar to each other and different from the starting structure.

## 4. Conclusions

The distance profiles of the single and double mutants in the unbound state confirm that the D60E and I62V mutations shift protein conformation to a higher closed conformation percentage. This increase was also observed in MD simulations. This result explains the most probable closed conformation distance of the F and H variants presented in our previous work [80]. MD simulations reveal a salt bridge interaction between D60E and K43 was facilitated in the presence of the D60E mutation to stabilize a closed flap conformation. I62V likely participates in a hydrophobic sliding mechanism, resulting in a change in conformation. Together with other NPs present in F and H variants, D60E and I62V indirectly contribute to conformational change and possibly affect binding to PIs other than DRV [80].

## Figures and Tables

**Table 1:** Summary of most probable nitroxide distances and fractional occupancy for HIV-1PR constructs.

	Most Probable Distance (±0.2 Å)	Conformation Population Percentages			
		Curled/Tucked	Closed	Semi-Open	Open
B ^a^	36.2	4 ± 3	3 ± 3	86 ± 3	7 ± 3
F ^b^	34.4	20 ± 4	49 ± 5	28 ± 5	3 ± 3
H ^b^	33.7	11 ± 5	48 ± 5	41 ± 5	0 ± 5
D60E	34.0	17 ± 5	65 ± 5	12 ± 5	7 ± 5
I62V	35.2	10 ± 3	49 ± 3	41 ± 3	0 ± 3
D60E/I63E	34.2 and 36.5	10 ± 4	59 ± 4	26 ± 4	5 ± 4
A71V ^c^	33.0	17 ± 6	67 ± 6	7 ± 6	9 ± 6
L/I63P ^d^	33.2	10 ± 5	57 ± 5	27 ± 5	6 ± 5

^a^ data taken from ref. [76]; ^b^ data taken from ref. [80]; ^c^ data taken from ref. [25]; ^d^ data taken from ref. [95].

## Data Availability

Data will be made available upon request. DEER distance analyses are provided within the supporting information.

## Supplementary Materials

The following supporting information can be downloaded at: https://www.mdpi.com/article/10.3390/v16020236/s1. Figure S1: DEER data processing for D60E-unbound. Figure S2: DEER data processing for the D60E-DRV. Figure S3: DEER data processing for I62V-unbound. Figure S4: DEER data processing for I62V-DRV. Figure S5: DEER data processing for D60E/I62V-unbound. Figure S6: DEER data processing for D60E/I62V-DRV. Figure S7: DEER distance profiles and populations with DRV. Figure S8: Distance profiles for flaps and flap tips in a closed conformation. Figure S9. Ribbon diagrams comparing the starting equilibrated structure (1HHP) in yellow and (A) average structures obtained from analysis of conformers sampled during the 1.6 μs simulation runs with (B) focusing on details of the flap tips showing that D60E and I62V flip their handedness during the simulations. Table S1: Analysis of flap and flap-tip distances from HIV-1 PR crystal structured deposited within the PDB.

## References

[1] Wensing A.M., Calvez V., Ceccherini-Silberstein F., Charpentier C., Gunthard H.F., Paredes R., Shafer R.W., Richman D.D. (2019). 2019 update of the drug resistance mutations in HIV-1. Top. Antivir. Med..

[2] Walensky R.P., Paltiel A.D., Losina E., Mercincavage L.M., Schackman B.R., Sax P.E., Weinstein M.C., Freedberg K.A. (2006). The survival benefits of AIDS treatment in the United States. J. Infect. Dis..

[3] Martinez-Cajas J.L., Wainberg M.A. (2007). Protease inhibitor resistance in HIV-infected patients: Molecular and clinical perspectives. Antivir. Res..

[4] Shafer R.W., Schapiro J.M. (2008). HIV-1 drug resistance mutations: An updated framework for the second decade of HAART. Aids Rev..

[5] Weber I.T., Agniswamy J. (2009). HIV-1 Protease: Structural Perspectives on Drug Resistance. Viruses.

[6] Kurt Yilmaz N., Swanstrom R., Schiffer C.A. (2016). Improving Viral Protease Inhibitors to Counter Drug Resistance. Trends Microbiol..

[7] Ghosh A.K., Osswald H.L., Prato G. (2016). Recent Progress in the Development of HIV-1 Protease Inhibitors for the Treatment of HIV/AIDS. J. Med. Chem..

[8] Weber I.T., Kneller D.W., Wong-Sam A. (2015). Highly resistant HIV-1 proteases and strategies for their inhibition. Future Med. Chem..

[9] Wlodawer A., Gustchina A. (2000). Structural and biochemical studies of retroviral proteases. Biochim. Biophys. Acta.

[10] Wlodawer A., Vondrasek J. (1998). Inhibitors of HIV-1 protease: A major success of structure-assisted drug design. Annu. Rev. Biophys. Biomol. Struct..

[11] (2010). UNAIDS Report on the Global AIDS Epidemic.

[12] Santos A.F., Soares M.A. (2010). HIV Genetic Diversity and Drug Resistance. Viruses.

[13] Ashorn P., McQuade T.J., Thaisrivongs S., Tomasselli A.G., Tarpley W.G., Moss B. (1990). An inhibitor of the protease blocks maturation of human and simian immunodeficiency viruses and spread of infection. Proc. Natl. Acad. Sci. USA.

[14] Sayer J.M., Liu F., Ishima R., Weber I.T., Louis J.M. (2008). Effect of the active site D25N mutation on the structure, stability, and ligand binding of the mature HIV-1 protease. J. Biol. Chem..

[15] Ishima R., Freedberg D.I., Wang Y.X., Louis J.M., Torchia D.A. (1999). Flap opening and dimer-interface flexibility in the free and inhibitor-bound HIV protease, and their implications for function. Structure.

[16] Heaslet H., Rosenfeld R., Giffin M., Lin Y.C., Tam K., Torbett B.E., Elder J.H., McRee D.E., Stout C.D. (2007). Conformational flexibility in the flap domains of ligand-free HIV protease. Acta Crystallogr. D Biol. Crystallogr..

[17] Spinelli S., Liu Q.Z., Alzari P.M., Hirel P.H., Poljak R.J. (1991). The three-dimensional structure of the aspartyl protease from the HIV-1 isolate BRU. Biochimie.

[18] Galiano L., Bonora M., Fanucci G.E. (2007). Inter-flap distances in HIV-1 Protease determined by pulsed EPR measurements. J. Am. Chem. Soc..

[19] Freedberg D.I., Ishima R., Jacob J., Wang Y.X., Kustanovich I., Louis J.M., Torchia D.A. (2002). Rapid structural fluctuations of the free HIV protease flaps in solution: Relationship to crystal structures and comparison with predictions of dynamics calculations. Protein Sci..

[20] Tozzini V., Trylska J., Chang C.E., McCammon J.A. (2007). Flap opening dynamics in HIV-1 protease explored with a coarse-grained model. J. Struct. Biol..

[21] Toth G., Borics A. (2006). Flap opening mechanism of HIV-1 protease. J. Mol. Graph. Model..

[22] Karthik S., Senapati S. (2011). Dynamic flaps in HIV-1 protease adopt unique ordering at different stages in the catalytic cycle. Proteins.

[23] Hornak V., Okur A., Rizzo R.C., Simmerling C. (2006). HIV-1 protease flaps spontaneously open and reclose in molecular dynamics simulations. Proc. Natl. Acad. Sci. USA.

[24] Ding F., Layten M., Simmerling C. (2008). Solution structure of HIV-1 protease flaps probed by comparison of molecular dynamics simulation ensembles and EPR experiments. J. Am. Chem. Soc..

[25] de Vera I.M., Smith A.N., Dancel M.C., Huang X., Dunn B.M., Fanucci G.E. (2013). Elucidating a relationship between conformational sampling and drug resistance in HIV-1 protease. Biochemistry.

[26] Kear J.L., Blackburn M.E., Veloro A.M., Dunn B.M., Fanucci G.E. (2009). Subtype polymorphisms among HIV-1 protease variants confer altered flap conformations and flexibility. J. Am. Chem. Soc..

[27] Liu Z., Casey T.M., Blackburn M.E., Huang X., Pham L., de Vera I.M., Carter J.D., Kear-Scott J.L., Veloro A.M., Galiano L. (2016). Pulsed EPR characterization of HIV-1 protease conformational sampling and inhibitor-induced population shifts. Phys. Chem. Chem. Phys..

[28] Deshmukh L., Louis J.M., Ghirlando R., Clore G.M. (2016). Transient HIV-1 Gag-protease interactions revealed by paramagnetic NMR suggest origins of compensatory drug resistance mutations. Proc. Natl. Acad. Sci. USA.

[29] Deshmukh L., Tugarinov V., Louis J.M., Clore G.M. (2017). Binding kinetics and substrate selectivity in HIV-1 protease-Gag interactions probed at atomic resolution by chemical exchange NMR. Proc. Natl. Acad. Sci. USA.

[30] Agniswamy J., Louis J.M., Roche J., Harrison R.W., Weber I.T. (2016). Structural Studies of a Rationally Selected Multi-Drug Resistant HIV-1 Protease Reveal Synergistic Effect of Distal Mutations on Flap Dynamics. PLoS ONE.

[31] Miller M., Schneider J., Sathyanarayana B.K., Toth M.V., Marshall G.R., Clawson L., Selk L., Kent S.B., Wlodawer A. (1989). Structure of complex of synthetic HIV-1 protease with a substrate-based inhibitor at 2.3 A resolution. Science.

[32] Weber I.T., Miller M., Jaskolski M., Leis J., Skalka A.M., Wlodawer A. (1989). Molecular modeling of the HIV-1 protease and its substrate binding site. Science.

[33] Clemente J.C., Coman R.M., Thiaville M.M., Janka L.K., Jeung J.A., Nukoolkarn S., Govindasamy L., Agbandje-McKenna M., McKenna R., Leelamanit W. (2006). Analysis of HIV-1 CRF_01 A/E protease inhibitor resistance: Structural determinants for maintaining sensitivity and developing resistance to atazanavir. Biochemistry.

[34] Martin P., Vickrey J.F., Proteasa G., Jimenez Y.L., Wawrzak Z., Winters M.A., Merigan T.C., Kovari L.C. (2005). “Wide-open” 1.3 A structure of a multidrug-resistant HIV-1 protease as a drug target. Structure.

[35] Coman R.M., Robbins A.H., Goodenow M.M., Dunn B.M., McKenna R. (2008). High-resolution structure of unbound human immunodeficiency virus 1 subtype C protease: Implications of flap dynamics and drug resistance. Acta Crystallogr. D Biol. Crystallogr..

[36] Shen C.H., Chang Y.C., Agniswamy J., Harrison R.W., Weber I.T. (2015). Conformational variation of an extreme drug resistant mutant of HIV protease. J. Mol. Graph. Model..

[37] Wong-Sam A., Wang Y.F., Kneller D.W., Kovalevsky A.Y., Ghosh A.K., Harrison R.W., Weber I.T. (2022). HIV-1 protease with 10 lopinavir and darunavir resistance mutations exhibits altered inhibition, structural rearrangements and extreme dynamics. J. Mol. Graph. Model..

[38] Souffrant M., Yao X.Q., Hamelberg D. (2023). Evolving Mutational Buildup in HIV-1 Protease Shifts Conformational Dynamics to Gain Drug Resistance. J. Chem. Inf. Model..

[39] Galiano L., Ding F., Veloro A.M., Blackburn M.E., Simmerling C., Fanucci G.E. (2009). Drug pressure selected mutations in HIV-1 protease alter flap conformations. J. Am. Chem. Soc..

[40] Johnson V.A., Calvez V., Gunthard H.F., Paredes R., Pillay D., Shafer R., Wensing A.M., Richman D.D. (2011). 2011 update of the drug resistance mutations in HIV-1. Top. Antivir. Med..

[41] Wensing A.M., Calvez V., Gunthard H.F., Johnson V.A., Paredes R., Pillay D., Shafer R.W., Richman D.D. (2014). 2014 Update of the drug resistance mutations in HIV-1. Top. Antivir. Med..

[42] Muzammil S., Armstrong A.A., Kang L.W., Jakalian A., Bonneau P.R., Schmelmer V., Amzel L.M., Freire E. (2007). Unique thermodynamic response of tipranavir to human immunodeficiency virus type 1 protease drug resistance mutations. J. Virol..

[43] Doyon L., Tremblay S., Bourgon L., Wardrop E., Cordingley M.G. (2005). Selection and characterization of HIV-1 showing reduced susceptibility to the non-peptidic protease inhibitor tipranavir. Antivir. Res..

[44] Sturmer M., Stephan C., Gute P., Knecht G., Bickel M., Brodt H.R., Doerr H.W., Gurtler L., Lecocq P., van Houtte M. (2011). Comparison of drug resistance scores for tipranavir in protease inhibitor-naive patients infected with HIV-1 B and non-B subtypes. Antimicrob. Agents Chemother..

[45] Nalam M.N., Schiffer C.A. (2008). New approaches to HIV protease inhibitor drug design II: Testing the substrate envelope hypothesis to avoid drug resistance and discover robust inhibitors. Curr. Opin. HIV AIDS.

[46] Wang Y., Liu Z., Brunzelle J.S., Kovari I.A., Dewdney T.G., Reiter S.J., Kovari L.C. (2011). The higher barrier of darunavir and tipranavir resistance for HIV-1 protease. Biochem. Biophys. Res. Commun..

[47] Buonaguro L., Tornesello M.L., Buonaguro F.M. (2007). Human immunodeficiency virus type 1 subtype distribution in the worldwide epidemic: Pathogenetic and therapeutic implications. J. Virol..

[48] Weber I.T., Wang Y.F., Harrison R.W. (2021). HIV Protease: Historical Perspective and Current Research. Viruses.

[49] Krauchenco S., Martins N.H., Sanches M., Polikarpov I. (2009). Effectiveness of commercial inhibitors against subtype F HIV-1 protease. J. Enzym. Inhib. Med. Chem..

[50] Mata-Munguia C., Escoto-Delgadillo M., Torres-Mendoza B., Flores-Soto M., Vazquez-Torres M., Galvez-Gastelum F., Viniegra-Osorio A., Castillero-Manzano M., Vazquez-Valls E. (2014). Natural polymorphisms and unusual mutations in HIV-1 protease with potential antiretroviral resistance: A bioinformatic analysis. BMC Bioinform..

[51] Holguin A., Sune C., Hamy F., Soriano V., Klimkait T. (2006). Natural polymorphisms in the protease gene modulate the replicative capacity of non-B HIV-1 variants in the absence of drug pressure. J. Clin. Virol..

[52] Holguin A., Paxinos E., Hertogs K., Womac C., Soriano V. (2004). Impact of frequent natural polymorphisms at the protease gene on the in vitro susceptibility to protease inhibitors in HIV-1 non-B subtypes. J. Clin. Virol..

[53] Agniswamy J., Sayer J.M., Weber I.T., Louis J.M. (2012). Terminal interface conformations modulate dimer stability prior to amino terminal autoprocessing of HIV-1 protease. Biochemistry.

[54] Chiang M., Wang C. (2021). A Single Amino Acid Substitution at the HIV-1 Protease Termini Dimer Interface Significantly Reduces Viral Particles Processing Efficiency. Jpn. J. Infect. Dis..

[55] Dakshinamoorthy A., Asmita A., Senapati S. (2023). Comprehending the Structure, Dynamics, and Mechanism of Action of Drug-Resistant HIV Protease. ACS Omega.

[56] Kim J.G., Shan L. (2022). Beyond Inhibition: A Novel Strategy of Targeting HIV-1 Protease to Eliminate Viral Reservoirs. Viruses.

[57] Okafor S.N., Angsantikul P., Ahmed H. (2022). Discovery of Novel HIV Protease Inhibitors Using Modern Computational Techniques. Int. J. Mol. Sci..

[58] Rahimi M., Taghdir M., Abasi Joozdani F. (2023). Dynamozones are the most obvious sign of the evolution of conformational dynamics in HIV-1 protease. Sci. Rep..

[59] Rajendran M., Ferran M.C., Mouli L., Babbitt G.A., Lynch M.L. (2023). Evolution of drug resistance drives destabilization of flap region dynamics in HIV-1 protease. Biophys. Rep..

[60] Sheik Ismail Z., Worth R., Mosebi S., Sayed Y. (2023). HIV Protease Hinge Region Insertions at Codon 38 Affect Enzyme Kinetics, Conformational Stability and Dynamics. Protein J..

[61] Spielvogel E., Lee S.K., Zhou S., Lockbaum G.J., Henes M., Sondgeroth A., Kosovrasti K., Nalivaika E.A., Ali A., Yilmaz N.K. (2023). Selection of HIV-1 for resistance to fifth-generation protease inhibitors reveals two independent pathways to high-level resistance. eLife.

[62] Tunc H., Dogan B., Darendeli Kiraz B.N., Sari M., Durdagi S., Kotil S. (2023). Prediction of HIV-1 protease resistance using genotypic, phenotypic, and molecular information with artificial neural networks. PeerJ.

[63] Wang Q., Gao H., Clark K.M., Mugisha C.S., Davis K., Tang J.P., Harlan G.H., DeSelm C.J., Presti R.M., Kutluay S.B. (2021). CARD8 is an inflammasome sensor for HIV-1 protease activity. Science.

[64] Yu F.H., Huang K.J., Wang C.T. (2021). Conditional activation of an HIV-1 protease attenuated mutant by a leucine zipper dimerization motif. Virus Res..

[65] Zhu M., Shan Q., Ma L., Dong B., Wang J., Zhang G., Wang M., Zhou J., Cen S., Wang Y. (2023). Structure based design and evaluation of benzoheterocycle derivatives as potential dual HIV-1 protease and reverse transcriptase inhibitors. Eur. J. Med. Chem..

[66] Lin K.H., Ali A., Rusere L., Soumana D.I., Kurt Yilmaz N., Schiffer C.A. (2017). Dengue Virus NS2B/NS3 Protease Inhibitors Exploiting the Prime Side. J. Virol..

[67] Lin K.H., Nalivaika E.A., Prachanronarong K.L., Yilmaz N.K., Schiffer C.A. (2016). Dengue Protease Substrate Recognition: Binding of the Prime Side. ACS Infect. Dis..

[68] Zephyr J., Kurt Yilmaz N., Schiffer C.A. (2021). Viral proteases: Structure, mechanism and inhibition. Enzymes.

[69] Borbat P.P., Freed J.H., Timmel C.R., Harmer J.R. (2014). Pulse Dipolar Electron Spin Resonance: Distance Measurements. Structural Information from Spin-Labels and Intrinsic Paramagnetic Centres in the Biosciences.

[70] Ghimire H., McCarrick R.M., Budil D.E., Lorigan G.A. (2009). Significantly improved sensitivity of Q-band PELDOR/DEER experiments relative to X-band is observed in measuring the intercoil distance of a leucine zipper motif peptide (GCN4-LZ). Biochemistry.

[71] Goldfarb D. (2022). Exploring protein conformations in vitro and in cell with EPR distance measurements. Curr. Opin. Struct. Biol..

[72] Jeschke G. (2012). DEER Distance Measurements on Proteins. Annu. Rev. Phys. Chem..

[73] Polyhach Y., Bordingnon E., Tschaggler S., Gandra A., Godt A., Jeschke G. (2012). High Sensitivity and versatality of the DEER experiment on nitroxide radical pairs at Q-band frequenies. Phys. Chem. Chem. Phys..

[74] Reginsson G.W., Schiemann O. (2011). Pulsed electron-electron double resonance: Beyond nanometre distance measurements on biomacromolecules. Biochem. J..

[75] Torricella F., Pierro A., Mileo E., Belle V., Bonucci A. (2021). Nitroxide spin labels and EPR spectroscopy: A powerful association for protein dynamics studies. Biochim. Biophys. Acta Proteins Proteom..

[76] Blackburn M.E., Veloro A.M., Fanucci G.E. (2009). Monitoring inhibitor-induced conformational population shifts in HIV-1 protease by pulsed EPR spectroscopy. Biochemistry.

[77] de Vera I.M., Blackburn M.E., Fanucci G.E. (2012). Correlating conformational shift induction with altered inhibitor potency in a multidrug resistant HIV-1 protease variant. Biochemistry.

[78] Huang X., de Vera I.M., Veloro A.M., Blackburn M.E., Kear J.L., Carter J.D., Rocca J.R., Simmerling C., Dunn B.M., Fanucci G.E. (2012). Inhibitor-induced conformational shifts and ligand-exchange dynamics for HIV-1 protease measured by pulsed EPR and NMR spectroscopy. J. Phys. Chem. B.

[79] Liu Z., Tran T.T., Pham L., Hu L., Bentz K., Savin D.A., Fanucci G.E. (2020). Darunavir-Resistant HIV-1 Protease Constructs Uphold a Conformational Selection Hypothesis for Drug Resistance. Viruses.

[80] Tran T.T., Liu Z., Fanucci G.E. (2020). Conformational landscape of non-B variants of HIV-1 protease: A pulsed EPR study. Biochem. Biophys. Res. Commun..

[81] Huang X., Britto M.D., Kear-Scott J.L., Boone C.D., Rocca J.R., Simmerling C., McKenna R., Bieri M., Gooley P.R., Dunn B.M. (2014). The role of select subtype polymorphisms on HIV-1 protease conformational sampling and dynamics. J. Biol. Chem..

[82] Liu Z., Huang X., Hu L., Pham L., Poole K.M., Tang Y., Mahon B.P., Tang W., Li K., Goldfarb N.E. (2016). Effects of Hinge-region Natural Polymorphisms on Human Immunodeficiency Virus-Type 1 Protease Structure, Dynamics, and Drug Pressure Evolution. J. Biol. Chem..

[83] Carter J.D., Gonzales E.G., Huang X., Smith A.N., de Vera I.M., D’Amore P.W., Rocca J.R., Goodenow M.M., Dunn B.M., Fanucci G.E. (2014). Effects of PRE and POST therapy drug-pressure selected mutations on HIV-1 protease conformational sampling. FEBS Lett..

[84] Mildner A.M., Rothrock D.J., Leone J.W., Bannow C.A., Lull J.M., Reardon I.M., Sarcich J.L., Howe W.J., Tomich C.S., Smith C.W. (1994). The HIV-1 protease as enzyme and substrate: Mutagenesis of autolysis sites and generation of a stable mutant with retained kinetic properties. Biochemistry.

[85] Kear J.L., Galiano L., Veloro A.M., Harris J., Busenlehner L.S., Fanucci G.E. (2011). Monitoring the autoproteolysis of HIV-1 Protease by Site-Directed Spin-Labeling and Electron Paramagnetic Resonance Spectroscopy. J. Biophys. Chem..

[86] Louis J.M., Clore G.M., Gronenborn A.M. (1999). Autoprocessing of HIV-1 protease is tightly coupled to protein folding. Nat. Struct. Biol..

[87] Shao W., Everitt L., Manchester M., Loeb D.D., Hutchison C.A., Swanstrom R. (1997). Sequence requirements of the HIV-1 protease flap region determined by saturation mutagenesis and kinetic analysis of flap mutants. Proc. Natl. Acad. Sci. USA.

[88] Jeschke G., Chechik V., Ionita P., Godt A., Zimmermann H., Banham J., Timmel C.R., Hilger D., Jung H. (2006). DeerAnalysis2006—A Comprehensive Software Package for Analyzing Pulsed ELDOR Data. Appl. Mag. Reson..

[89] Casey T.M., Fanucci G.E. (2015). Spin labeling and Double Electron-Electron Resonance (DEER) to Deconstruct Conformational Ensembles of HIV Protease. Methods Enzym..

[90] de Vera I.M.S., Blackburn M.E., Galiano L., Fanucci G.E. (2013). Pulsed EPR distance measurements in soluble proteins by site-directed spin labeling (SDSL). Curren Protoc. Protein Sci..

[91] Chakravorty D.K., Hammes-Schiffer S. (2010). Impact of mutation on proton transfer reactions in ketosteroid isomerase: Insights from molecular dynamics simulations. J. Am. Chem. Soc..

[92] Chakravorty D.K., Wang B., Ucisik M.N., Merz K.M. (2011). Insight into the cation-pi interaction at the metal binding site of the copper metallochaperone CusF. J. Am. Chem. Soc..

[93] Chakravorty D.K., Wang B., Lee C.W., Giedroc D.P., Merz K.M. (2012). Simulations of allosteric motions in the zinc sensor CzrA. J. Am. Chem. Soc..

[94] Chakravorty D.K., Parker T.M., Guerra A.J., Sherrill C.D., Giedroc D.P., Merz K.M. (2013). Energetics of zinc-mediated interactions in the allosteric pathways of metal sensor proteins. J. Am. Chem. Soc..

[95] Carter J.D., Mathias J.D., Gomez E.F., Ran Y., Xu F., Galiano L., Tran N.Q., D’Amore P.W., Wright C.S., Chakravorty D.K. (2014). Characterizing solution surface loop conformational flexibility of the GM2 activator protein. J. Phys. Chem. B.

[96] Case D.A., Cheatham T.E., Darden T., Gohlke H., Luo R., Merz K.M., Onufriev A., Simmerling C., Wang B., Woods R.J. (2005). The Amber biomolecular simulation programs. J. Comput. Chem..

[97] Hornak V., Abel R., Okur A., Strockbine B., Roitberg A., Simmerling C. (2006). Comparison of multiple Amber force fields and development of improved protein backbone parameters. Proteins.

[98] Gordon J.C., Myers J.B., Folta T., Shoja V., Heath L.S., Onufriev A. (2005). H++: A server for estimating pKas and adding missing hydrogens to macromolecules. Nucleic Acids Res..

[99] Joung I.S., Cheatham T.E. (2008). Determination of alkali and halide monovalent ion parameters for use in explicitly solvated biomolecular simulations. J. Phys. Chem. B.

[100] Jorgensen W.L., Chandrasekhar J., Madura J.D., Impey R.W., Klein M.L. (1983). Comparison of Simple Potential Functions for Simulating Liquid Water. J. Chem. Phys..

[101] Adelman S.A., Doll J.D. (1976). Generalized Langevin Equation Approach for Atom/Solid-Surface Scattering: General Formulation for Classical Scattering off Harmonic Solids. J. Chem. Phys..

[102] Doll J.D., Dion D.R. (1976). Generalized Langevin Equation Approach for Atom/Solid Surface Scattering: Numerical Techniques for Gaussian Generalized Langevin Dynamics. J. Chem. Phys..

[103] Allen M.P., Tildesley D.J. (1987). Computer Simulations of Liquids.

[104] Clemente J.C., Hemrajani R., Blum L.E., Goodenow M.M., Dunn B.M. (2003). Secondary mutations M36I and A71V in the human immunodeficiency virus type 1 protease can provide an advantage for the emergence of the primary mutation D30N. Biochemistry.

[105] Clemente J.C., Moose R.E., Hemrajani R., Whitford L.R., Govindasamy L., Reutzel R., McKenna R., Agbandje-McKenna M., Goodenow M.M., Dunn B.M. (2004). Comparing the accumulation of active- and nonactive-site mutations in the HIV-1 protease. Biochemistry.

[106] Foulkes-Murzycki J.E., Scott W.R., Schiffer C.A. (2007). Hydrophobic sliding: A possible mechanism for drug resistance in human immunodeficiency virus type 1 protease. Structure.

[107] Goldfarb N.E., Ohanessian M., Biswas S., McGee T.D., Mahon B.P., Ostrov D.A., Garcia J., Tang Y., McKenna R., Roitberg A. (2014). Defective hydrophobic sliding mechanism and active site expansion in HIV-1 protease drug resistant variant Gly48Thr/Leu89Met: Mechanisms for the loss of saquinavir binding potency. Biochemistry.

[108] Mittal S., Cai Y., Nalam M.N., Bolon D.N., Schiffer C.A. (2012). Hydrophobic core flexibility modulates enzyme activity in HIV-1 protease. J. Am. Chem. Soc..

[109] Pillai B., Kannan K.K., Hosur M.V. (2001). 1.9 A X-ray study shows closed flap conformation in crystals of tethered HIV-1 PR. Proteins.

[110] Robbins A.H., Coman R.M., Bracho-Sanchez E., Fernandez M.A., Gilliland C.T., Li M., Agbandje-McKenna M., Wlodawer A., Dunn B.M., McKenna R. (2010). Structure of the unbound form of HIV-1 subtype A protease: Comparison with unbound forms of proteases from other HIV subtypes. Acta Crystallogr. D Biol. Crystallogr..

