# Supplementary material for: Natural Polymorphisms D60E and I62V Stabilize a Closed Conformation in HIV-1 Protease in the Absence of an Inhibitor or Substrate
Source: Viruses. 2024 Feb 2;16(2):236. doi: 10.3390/v16020236 (PMC10892587; doi:10.3390/v16020236)
Supplement: Supplementary file 1 [file viruses-16-00236-s001.zip › viruses-2789738-supplementary.pdf]

Supporting Information

Effects of Secondary Mutations D60E and I62V in HIV-1 Protease on Protein Conformation: a DEER and MD study

Trang T. Tran and Gail E. Fanucci*

Department of Chemistry, University of Florida, Gainesville, FL 32611

* Corresponding author

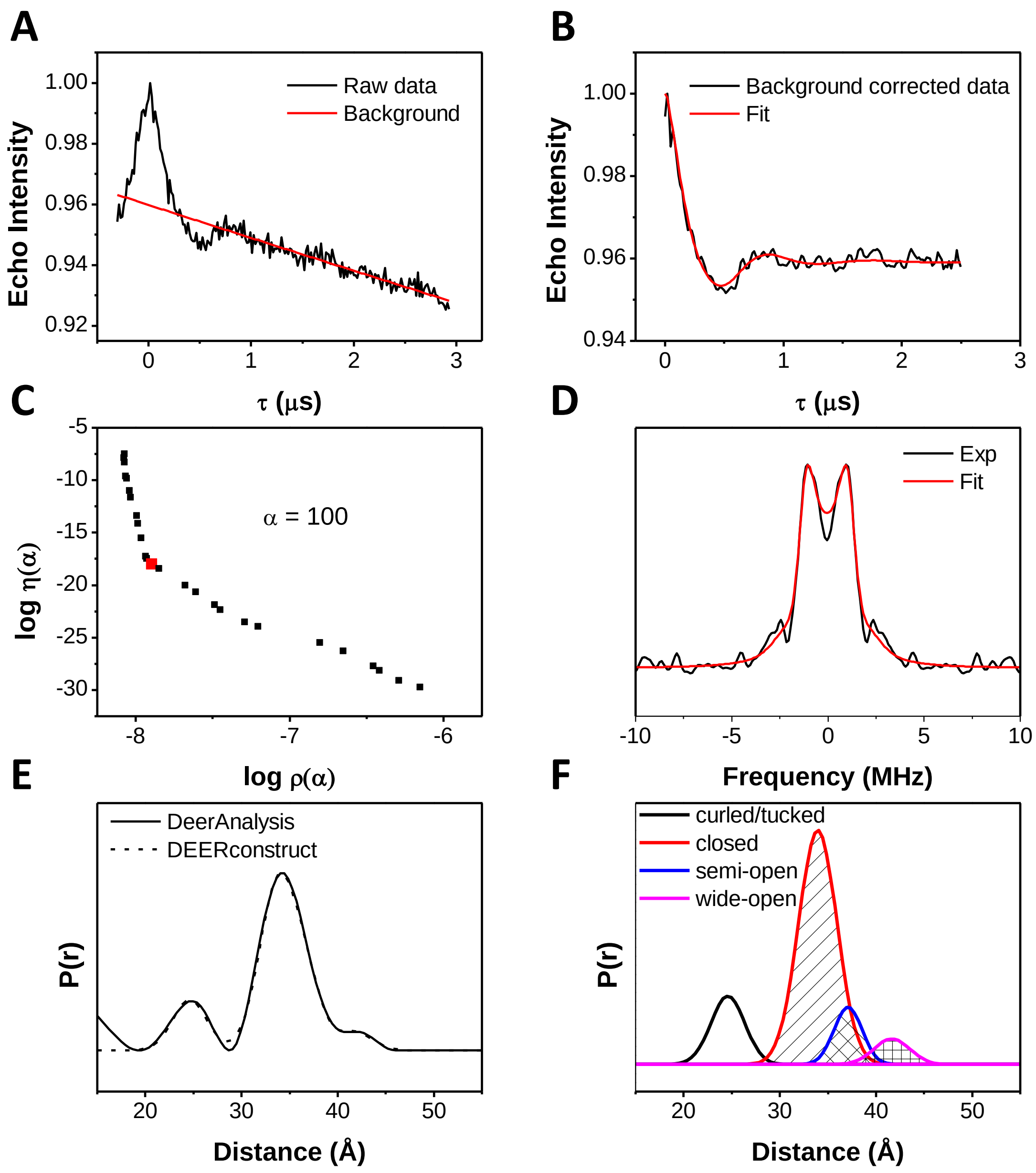

Figure S1. DEER data processing for D60E-unbound. (A) Raw dipolar echo curve with the corresponding exponential decay function used for background correction. (B) Background corrected dipolar modulation curve with the Tikhonov regularization (TKR) fit. (C) L-curve obtained from the TKR analysis with the corresponding optimal regularization parameter (α) enlarged and shown in red. (D) Pake pattern from the Fourier transformation of the background corrected dipolar modulation curve. (E) The TKR distance profile obtained (solid) and the reconstructed distance profile after population analysis and peak suppression (dash). (F) Individual Gaussian peaks used for reconstruction. Analysis follows procedures described in detail elsewhere.[1-3]

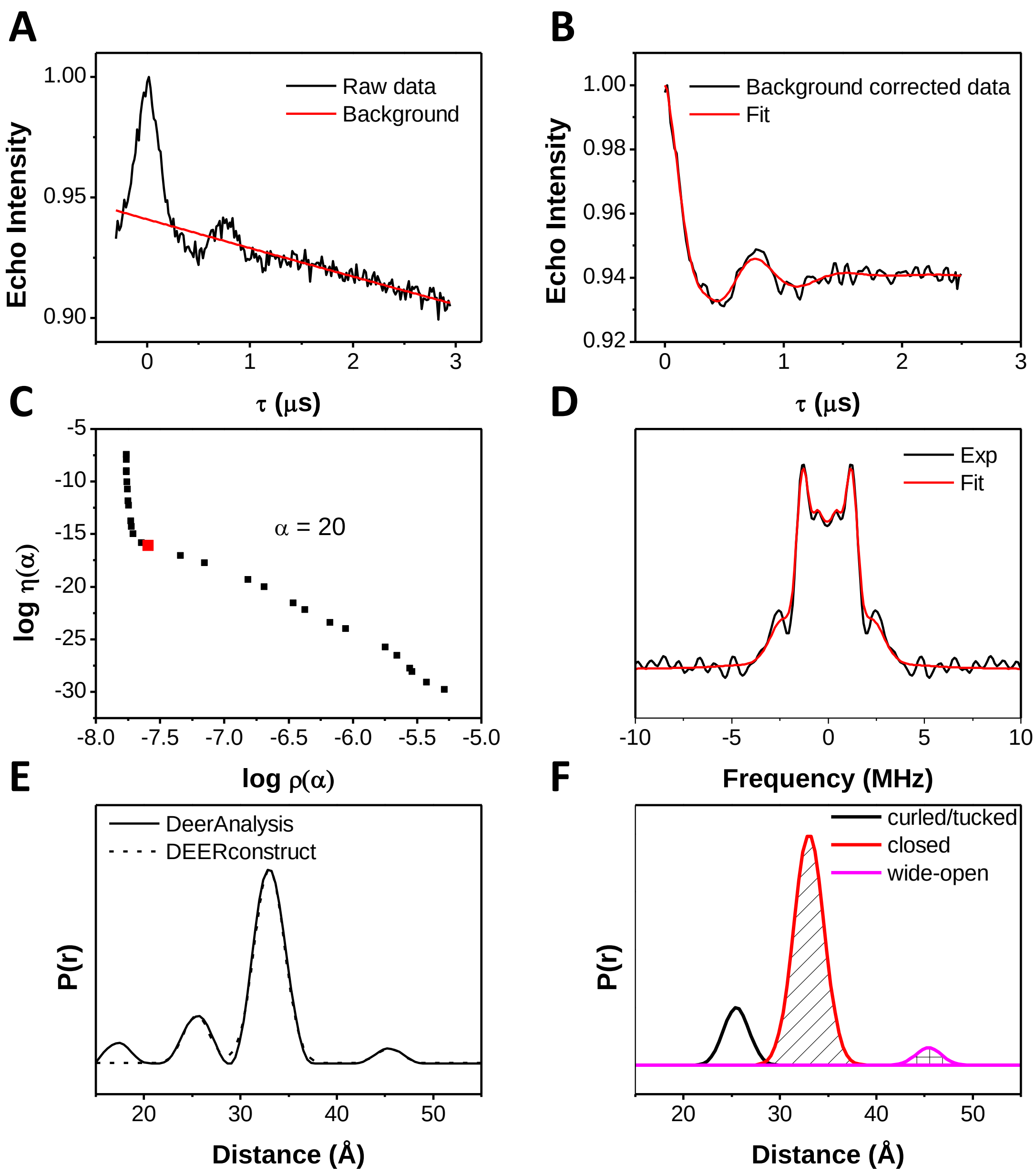

Figure S2. DEER data processing for D60E-DRV. (A) Raw dipolar echo curve with the corresponding exponential decay function used for background correction. (B) Background corrected dipolar modulation curve with the Tikhonov regularization (TKR) fit. (C) L-curve obtained from the TKR analysis with the corresponding optimal regularization parameter (α) enlarged and shown in red. (D) Pake pattern from the Fourier transformation of the background corrected dipolar modulation curve. (E) The TKR distance profile obtained (solid) and the reconstructed distance profile after population analysis and peak suppression (dash). (F) Individual Gaussian peaks used for reconstruction. Analysis follows procedures described in detail elsewhere.[1-3]

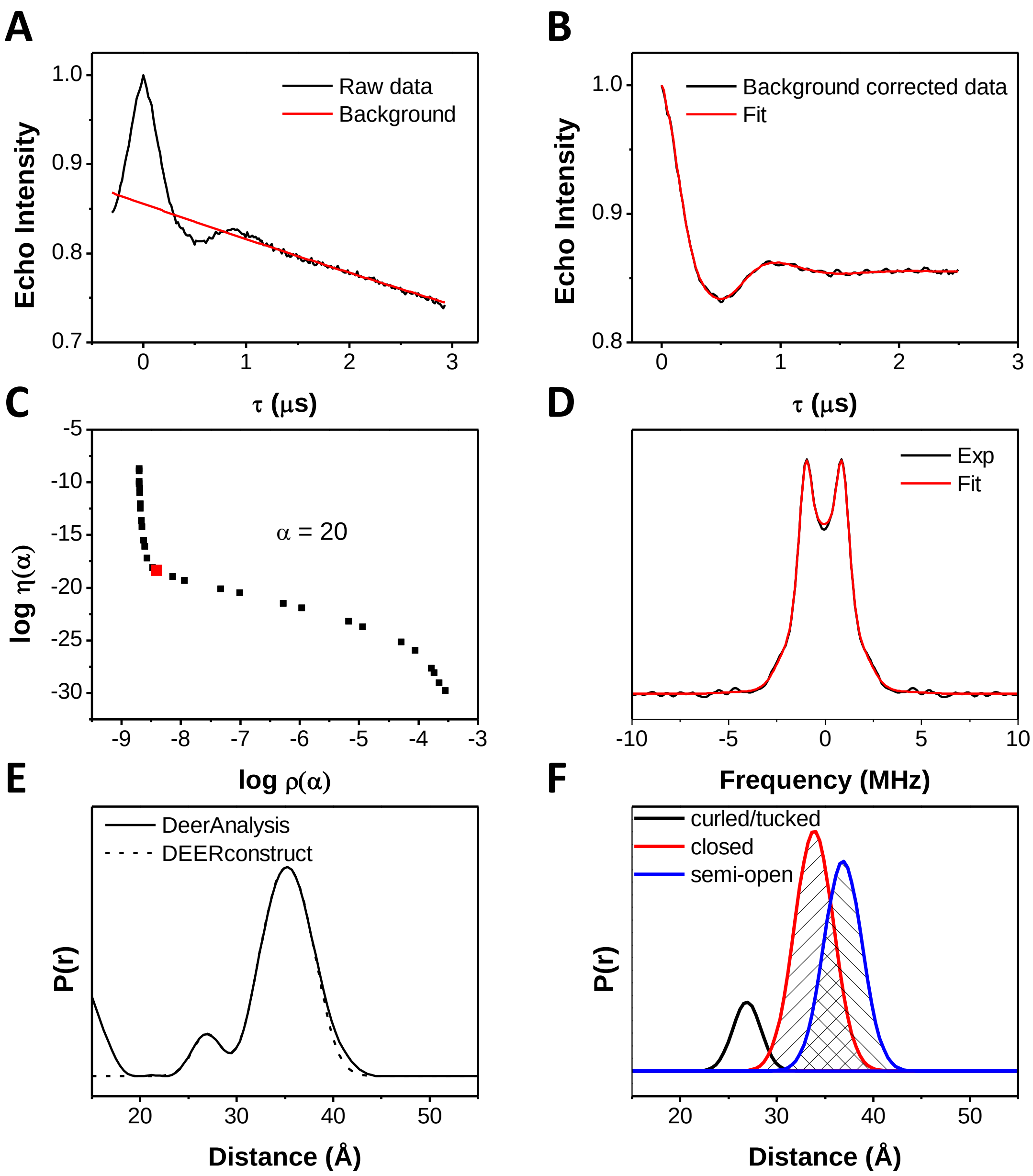

Figure S3. DEER data processing for I62V-unbound. (A) Raw dipolar echo curve with the corresponding exponential decay function used for background correction. (B) Background corrected dipolar modulation curve with the Tikhonov regularization (TKR) fit. (C) L-curve obtained from the TKR analysis with the corresponding optimal regularization parameter (α) enlarged and shown in red. (D) Pake pattern from the Fourier transformation of the background corrected dipolar modulation curve. (E) The TKR distance profile obtained (solid) and the reconstructed distance profile after population analysis and peak suppression (dash). (F) Individual Gaussian peaks used for reconstruction. Analysis follows procedures described in detail elsewhere.[1-3]

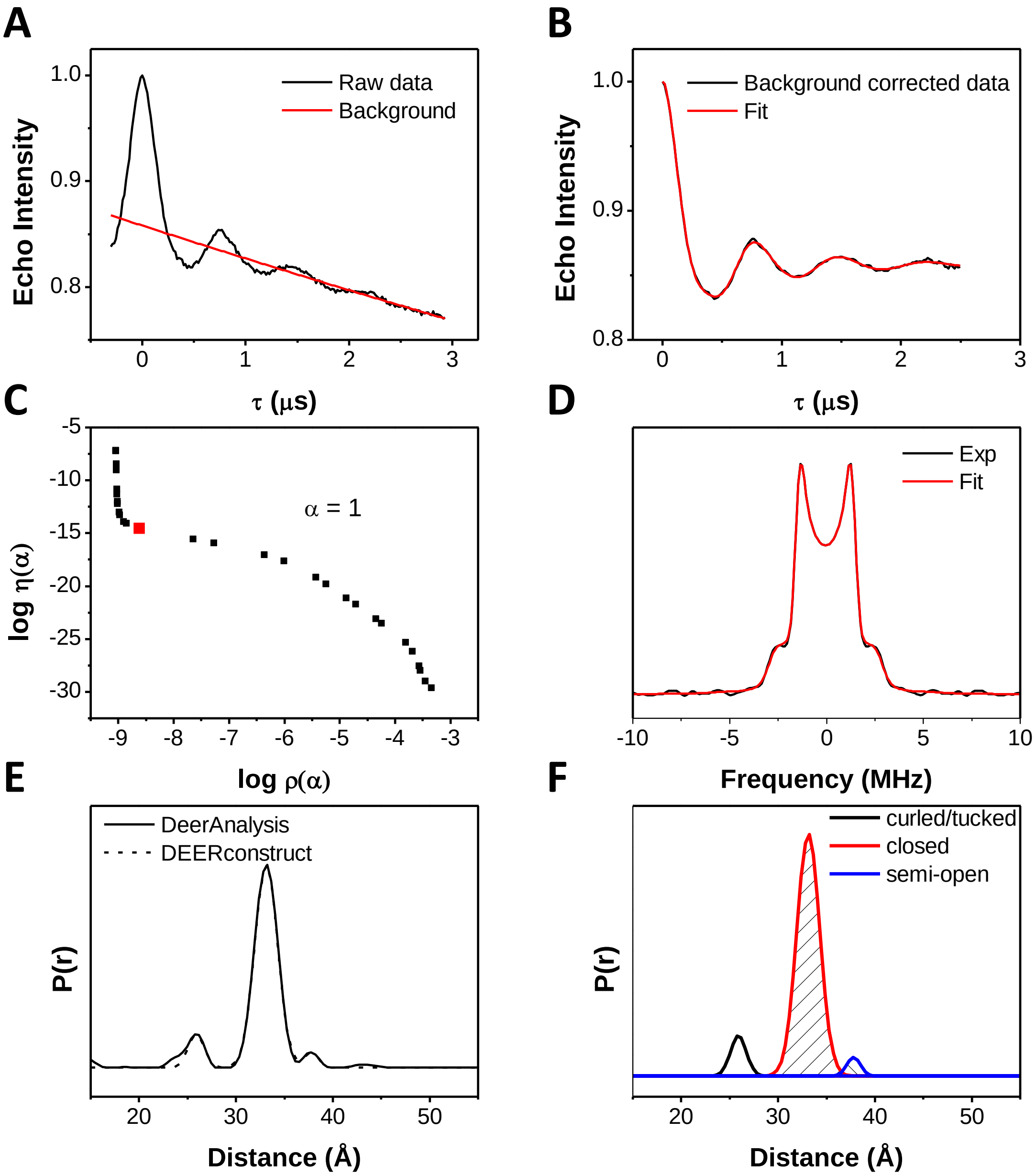

Figure S4. DEER data processing for I62V-DRV. (A) Raw dipolar echo curve with the corresponding exponential decay function used for background correction. (B) Background corrected dipolar modulation curve with the Tikhonov regularization (TKR) fit. (C) L-curve obtained from the TKR analysis with the corresponding optimal regularization parameter (α) enlarged and shown in red. (D) Pake pattern from the Fourier transformation of the background corrected dipolar modulation curve. (E) The TKR distance profile obtained (solid) and the reconstructed distance profile after population analysis and peak suppression (dash). (F) Individual Gaussian peaks used for reconstruction. Analysis follows procedures described in detail elsewhere.[1-3]

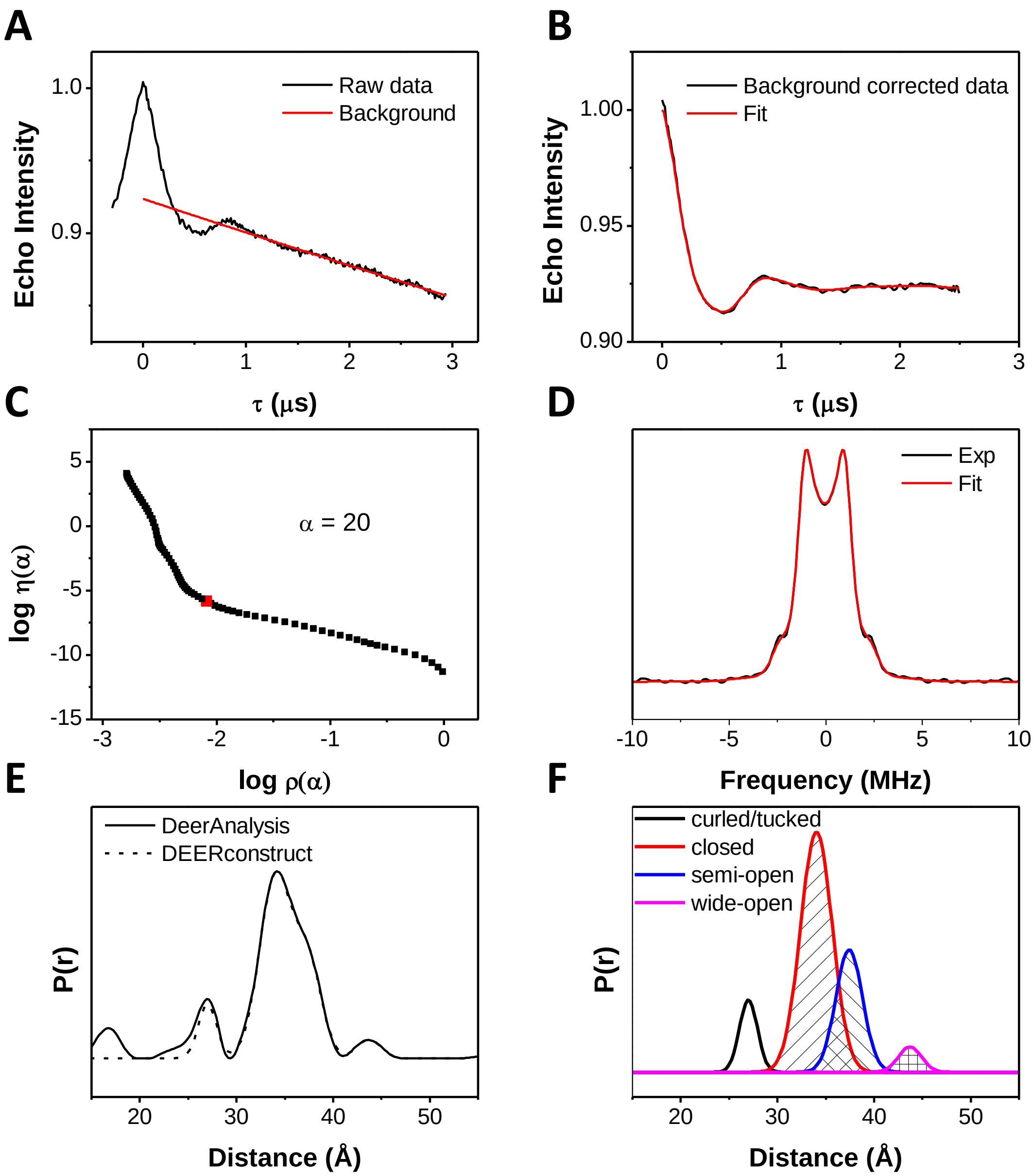

Figure S5. DEER data processing for D60E/I62V-unbound. (A) Raw dipolar echo curve with the corresponding exponential decay function used for background correction. (B) Background corrected dipolar modulation curve with the Tikhonov regularization (TKR) fit. (C) L-curve obtained from the TKR analysis with the corresponding optimal regularization parameter (α) enlarged and shown in red. (D) Pake pattern from the Fourier transformation of the background corrected dipolar modulation curve. (E) The TKR distance profile obtained (solid) and the reconstructed distance profile after population analysis and peak suppression (dash). (F) Individual Gaussian peaks used for reconstruction. Analysis follows procedures described in detail elsewhere.[1-3]

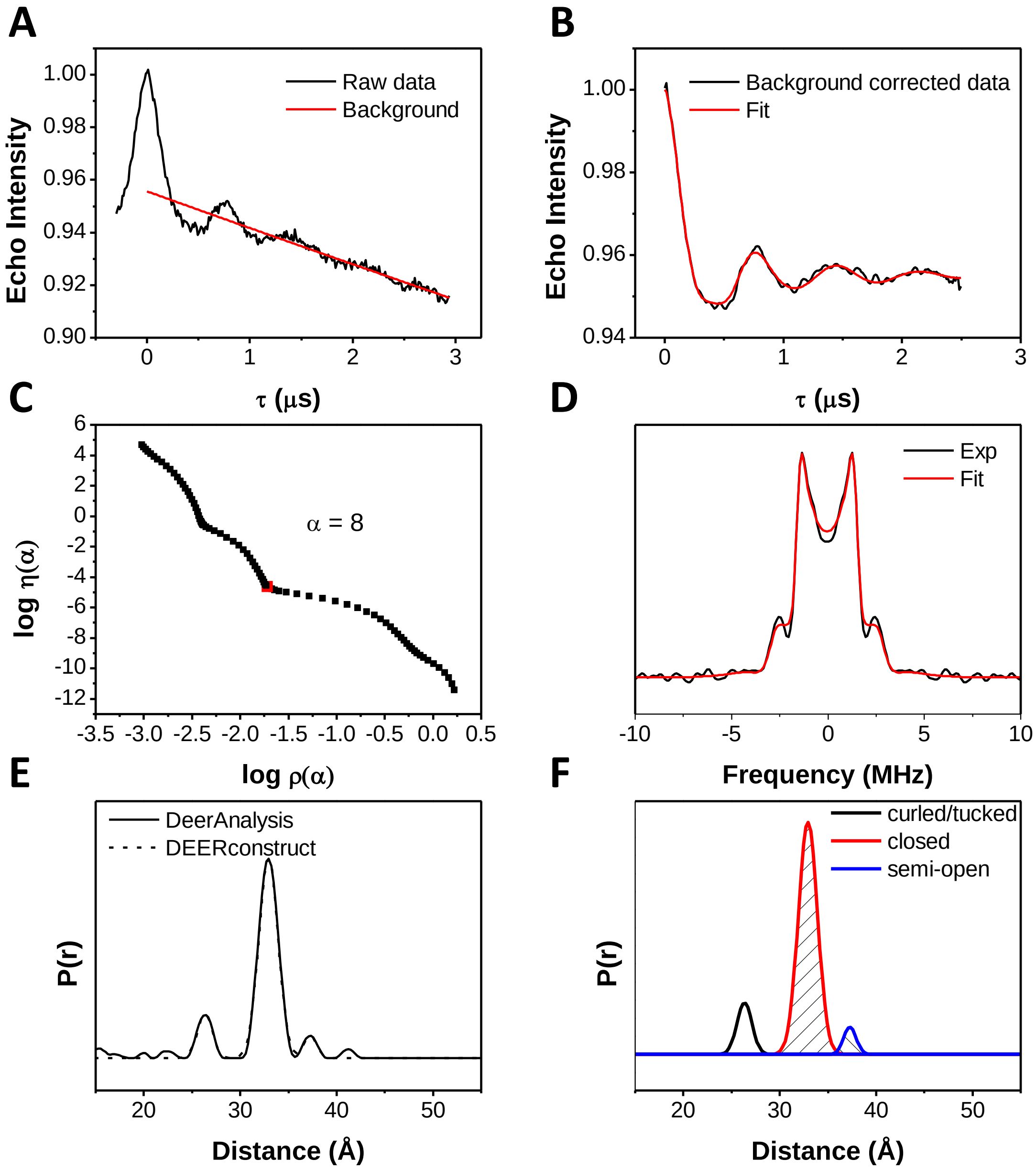

Figure S6. DEER data processing for D60E/I62V-DRV. (A) Raw dipolar echo curve with the corresponding exponential decay function used for background correction. (B) Background corrected dipolar modulation curve with the Tikhonov regularization (TKR) fit. (C) L-curve obtained from the TKR analysis with the corresponding optimal regularization parameter (α) enlarged and shown in red. (D) Pake pattern from the Fourier transformation of the background corrected dipolar modulation curve. (E) The TKR distance profile obtained (solid) and the reconstructed distance profile after population analysis and peak suppression (dash). (F) Individual Gaussian peaks used for reconstruction. Analysis follows procedures described in detail elsewhere.[1-3]

Inhibitors were obtained from the NIH AIDS Research and Reference Reagent Program, Division of AIDS, NIAID, NIH and the non-hydrolyzable CaP2 substrate mimic (H-Arg-Val-Leu-r-Phe-Glu-Ala-Nle/NH2 (r = reduced)) was purchased from Peptides International (KY). Inhibitors were dissolved in appropriate solvents[4-6] and were added to the protein samples in a 4:1 molar excess and allowed to incubate for 1 hour at room temperature prior to adding D8 glycerol to give a 30% v/v sample with 100 μM protein concentration.

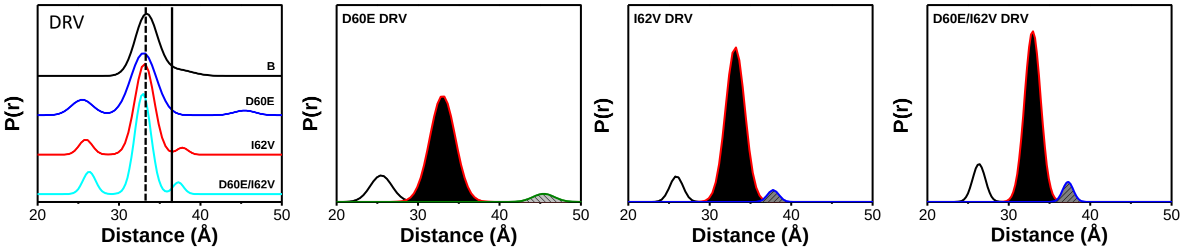

Figure S7. DEER distance profiles and Gaussian population analyses for DRV bound constructs showing that each construct adopted a closed conformation with DRV bound, but also demonstrated the presence of curled/tucked and wide-open flaps.

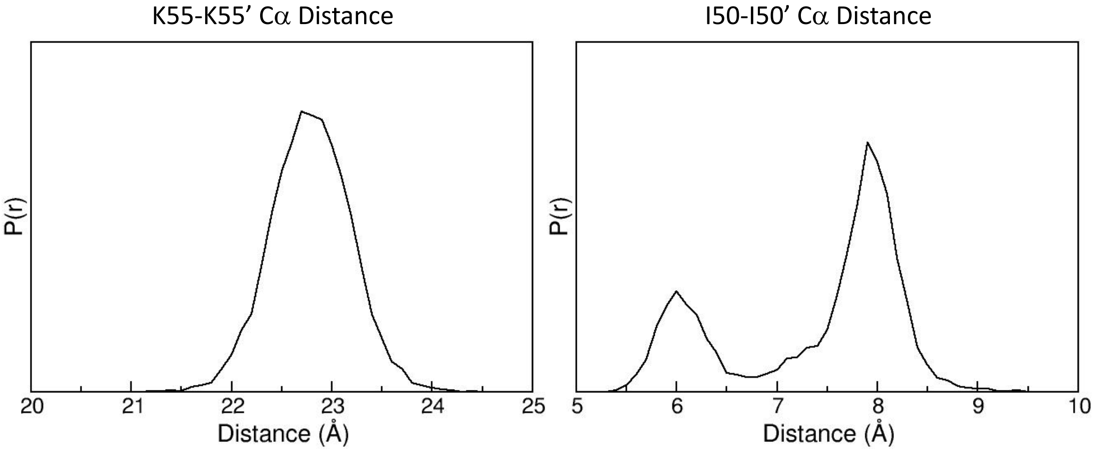

Figure S8. Distance profiles for flaps and flap tips in a closed conformation. (left) Flap distance (K55-K55′ Cα) and (fight) flap tip (I50-I50′ Cα) distances as probes for conformation in simulations determined from 1.6 microS simulation runs using PDB ID:3BVB of Darunavir bound HIV-1PR as the coordinates. During the time course of the simulations the flaps did not open.

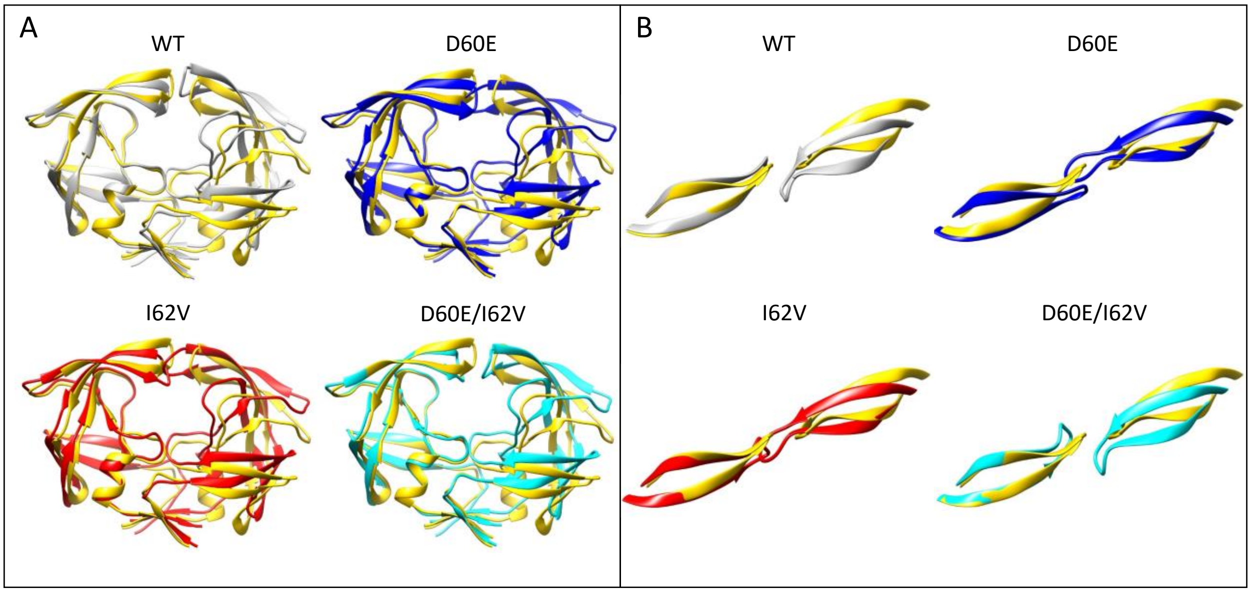

Figure S9. Ribbon diagrams comparing the starting equilibrated structure (1HHP) in yellow and (A) average structures obtained from analysis of conformers sampled during the 1.6 μs simulation runs with (B) focusing on details of the flap tips showing that D60E and I62V flip their handedness during the simulations.

Table S-1. Analysis of distances from HIV-1 PR crystal structured deposited within the PDB.[7-12]

Flap Conformation	PDB Code	Distances		
		K55-K55' Nζ (Å)	K55-K55' Cα (Å)	I50-I50' Cα (Å)
Closed	2P3C	19.9	22.4	6.0
	1G6L	29.8	22.3	6.0
	3IXO	28.1	22.1	5.9
Wide-open	2PC0	35.4	27.5	10.9
	1TW7	36.6	28.6	12.3
	2R8N	34.8	27.6	12.2
Semi-open	1HHP	33.1	27.1	4.3
	2HB4	33.0	26.1	4.6

[1] Casey, T. M., and Fanucci, G. E. (2015) Spin labeling and Double Electron-Electron Resonance (DEER) to Deconstruct Conformational Ensembles of HIV Protease, *Methods Enzymol 564*, 153-187.
[2] Liu, Z., Casey, T. M., Blackburn, M. E., Huang, X., Pham, L., de Vera, I. M., Carter, J. D., Kear-Scott, J. L., Veloro, A. M., Galiano, L., and Fanucci, G. E. (2016) Pulsed EPR characterization of HIV-1 protease conformational sampling and inhibitor-induced population shifts, *Phys Chem Chem Phys 18*, 5819-5831.
[3] de vera, I. M. S., Blackburn, M. E., Galiano, L., and Fanucci, G. E. (2013) Pulsed EPR distance measurements in soluble proteins by site-directed spin labeling (SDSL), *Curren Protocols in Protein Science 74*, 29.
[4] Tran, T. T., Liu, Z., and Fanucci, G. E. (2020) Conformational landscape of non-B variants of HIV-1 protease: A pulsed EPR study, *Biochem Biophys Res Commun 532*, 219-224.
[5] Blackburn, M. E., Veloro, A. M., and Fanucci, G. E. (2009) Monitoring inhibitor-induced conformational population shifts in HIV-1 protease by pulsed EPR spectroscopy, *Biochemistry 48*, 8765-8767.
[6] Huang, X., de Vera, I. M., Veloro, A. M., Blackburn, M. E., Kear, J. L., Carter, J. D., Rocca, J. R., Simmerling, C., Dunn, B. M., and Fanucci, G. E. (2012) Inhibitor-induced conformational shifts and ligand-exchange dynamics for HIV-1 protease measured by pulsed EPR and NMR spectroscopy, *J Phys Chem B 116*, 14235-14244.
[7] Krauchenco, S., Martins, N. H., Sanches, M., and Polikarpov, I. (2009) Effectiveness of commercial inhibitors against subtype F HIV-1 protease, *J Enzyme Inhib Med Chem 24*, 638-645.
[8] Pillai, B., Kannan, K. K., and Hosur, M. V. (2001) 1.9 A x-ray study shows closed flap conformation in crystals of tethered HIV-1 PR, *Proteins 43*, 57-64.
[9] Robbins, A. H., Coman, R. M., Bracho-Sanchez, E., Fernandez, M. A., Gilliland, C. T., Li, M., Agbandje-McKenna, M., Wlodawer, A., Dunn, B. M., and McKenna, R. (2010) Structure of the unbound form of HIV-1 subtype A protease: comparison with unbound forms of proteases from other HIV subtypes, *Acta Crystallogr D Biol Crystallogr 66*, 233-242.
[10] Heaslet, H., Rosenfeld, R., Giffin, M., Lin, Y. C., Tam, K., Torbett, B. E., Elder, J. H., McRee, D. E., and Stout, C. D. (2007) Conformational flexibility in the flap domains of ligand-free HIV protease, *Acta Crystallogr D Biol Crystallogr 63*, 866-875.
[11] Martin, P., Vickrey, J. F., Proteasa, G., Jimenez, Y. L., Wawrzak, Z., Winters, M. A., Merigan, T. C., and Kovari, L. C. (2005) "Wide-open" 1.3 A structure of a multidrug-resistant HIV-1 protease as a drug target, *Structure 13*, 1887-1895.
[12] Coman, R. M., Robbins, A. H., Goodenow, M. M., Dunn, B. M., and McKenna, R. (2008) High-resolution structure of unbound human immunodeficiency virus 1 subtype C protease: implications of flap dynamics and drug resistance, *Acta Crystallogr D Biol Crystallogr D64*, 754-763.
